# High TNF and NF-κB Pathway Dependency Are Associated with AZD5582 Sensitivity in OSCC via CASP8-Dependent Apoptosis

**DOI:** 10.1158/2767-9764.CRC-24-0136

**Published:** 2024-11-11

**Authors:** Annie Wai Yeeng Chai, Yee Hua Tan, Shiyin Ooi, Pei San Yee, Shi Mun Yee, Howard Lightfoot, Syd Barthorpe, Mathew J. Garnett, Sok Ching Cheong

**Affiliations:** 1 Translational Cancer Biology Research Unit, Cancer Research Malaysia, Subang Jaya, Malaysia.; 2 Wellcome Sanger Institute, Wellcome Genome Campus, Cambridge, United Kingdom.; 3 Faculty of Dentistry, University of Malaya, Kuala Lumpur, Malaysia.

## Abstract

Drug repurposing can accelerate the development of novel therapeutic strategies. Oral squamous cell carcinoma (OSCC) is prevalent in Asians and is associated with poor prognosis. In this study, we integrate newly generated sensitivity data for 339 anticancer drugs with genome-wide CRISPR-Cas9 gene essentiality data for 21 cell lines mostly derived from Asian patients with OSCC to identify drug repurposing candidates. We observe potent activity of AZD5582 (an antagonist of the inhibitor of apoptosis family of proteins) in a subset of OSCC cells, which was associated with dependency on NF-κB pathway genes (*RNF31*, *MAP3K7*, and *IKBKG*). We confirm the on-target specificity and potency of AZD5582 *in vitro* and its ability to suppress cell growth in xenograft models. Furthermore, we identify TNF as a key mediator of AZD5582 sensitivity in OSCC. Caspase-8–dependent apoptotic and Caspase-8–independent necroptotic cellular programs mediate AZD5582-induced cell death. In summary, through the systematic integration of pharmacologic and CRISPR data, we identified a subset of OSCC with potent sensitivity to AZD5582 mediated through the NF-κB and TNF signaling pathways.

**Significance:**

Mechanistically guided drug repurposing has been made possible by systematically integrating pharmacologic and CRISPR-Cas9 screen data. Our study discovers the biomarker and cell death mechanisms underpinning sensitivity toward AZD5582, an antagonist of the inhibitor of apoptosis family protein. Our findings have important implications for improving future trial design for patients with OSCC using this emerging drug class.

## Introduction

The integration of drug sensitivity data with genome-wide CRISPR-Cas9 screens has enabled the elucidation of the mechanism of action of drugs and has afforded opportunities to investigate potential markers underpinning drug sensitivity (1). Patients with oral squamous cell carcinoma (OSCC), a subtype of head and neck cancer (HNC) associated with poor survival, have limited opportunities for targeted therapy owing to the lack of actionable mutations. Notably, Asians make up more than 66% of worldwide OSCC cases, of which approximately 258,440 new incidences were diagnosed in Asia (2). For South Asian countries like India and Pakistan, OSCC is the commonest cancer and has the highest mortality rate among men (2). Tobacco use, alcohol consumption, and betel quid chewing are the major contributing risk factors toward OSCC. Surgery remains the primary treatment strategy for patients with OSCC, and for patients with unresectable tumor or those with high risk of recurrence, radiotherapy and chemoradiotherapy (CRT) are commonly used as adjuvant (3, 4). Since 2006, cetuximab (a mAb targeting the EGFR) remains the only approved molecular-targeted therapy. In 2016, two other immune checkpoint inhibitors, pembrolizumab and nivolumab, were subsequently approved as the second-line treatment for patients with cisplatin-refractory recurrent and metastatic OSCC, with pembrolizumab later being approved as the first-line therapy for those with unresectable or metastatic OSCC (4). However, limited responses and affordability/accessibility issues with these immune checkpoint inhibitors underscore the need to develop new treatments for patients with OSCC.

We previously conducted a genome-wide CRISPR-Cas9 screen on primarily Asian-derived OSCC cell lines and identified several promising targetable fitness genes (5). Notably, Asian-derived OSCCs associated with betel quid chewing are more dependent on the NF-κB signaling pathway, representing a potentially unique vulnerability to target this population (5). To accelerate new treatments to target these vulnerabilities, a drug repurposing approach is highly feasible, as many of the identified fitness genes are involved in oncogenic pathways targeted in other cancers. However, there is a paucity of drug sensitivity data for Asian-derived OSCC cell lines, among which the disease is the most prevalent. Furthermore, epidemiologic and genomic analyses of Asian and Caucasian OSCCs have revealed distinctive etiologies and genomic features (6). Thus, investigations of therapeutic responses using OSCC models derived from Asian patients are needed.

In this study, we screened 339 compounds on 21 OSCC cell lines derived primarily from Asian patients and integrated drug sensitivity data with CRISPR screen data to identify mechanistically informed drug repurposing candidates. Among the top hits, we observed potent sensitivity to AZD5582, a small-molecule antagonist of the inhibitor of apoptosis (IAP) family of proteins, which was correlated with fitness genes from the NF-κB pathway.

Overexpression of IAPs was reported in OSCC (7–9) and correlated with poor prognosis and pathologic response toward CRT (9). Preclinical studies using IAP inhibitors such as AZD5582 (10), LCL161 (11), birinapant (12), and xevinapant (Debio 1143; ref. 13) have demonstrated their efficacies in overcoming apoptosis evasion in HNC cell lines, especially when combined with radiotherapy. However, these studies have not delineated the exact mechanism of action of the drugs or investigated the differential genomic context of OSCC that gives rise to heterogeneous sensitivity. In this study, we report the binomial pattern of drug sensitivity with preferential potency of AZD5582 in OSCCs that are dependent on the NF-κB pathway genes. Furthermore, we also show that high TNF levels and functional caspase-8 (CASP8) expression were associated with AZD5582 sensitivity. Under TNF stimulation, the degradation of cIAP1/2 by AZD5582 leads to the release of RIP1 to form a complex with FADD and CASP8, activating apoptotic cell death (14). In CASP8 deficiency, RIP1 forms a necroptotic complex with RIP3 and MLKL, whereby phosphorylated MLKL induces cell death through necroptosis (14). Our study demonstrates the utility of integrative drug and CRISPR screen to identify and mechanistically assign AZD5582 sensitivity in a cohort of patients with OSCC.

## Materials and Methods

### Cell lines

All OSCC cell lines (ORL-48: CVCL_S692; ORL-115: CVCL_S690; ORL-136: CVCL_S691; ORL-150: CVCL_VJ37; ORL-153: CVCL_VJ38; ORL-156: CVCL_VJ39; ORL-166: CVCL_VJ40; ORL-174: CVCL_VJ41; ORL-188: CVCL_VJ42; ORL-195: CVCL_VJ43; ORL-204: CVCL_VJ45; ORL-207: CVCL_VJ46; ORL-214: CVCL_VJ47; ORL-215: CVCL_VJ48; BICR10: CVCL_2307; HSC-2: CVCL_1287; HSC-4: CVCL_1289; Ho-1-u-1: CVCL_2784; PE/CA-PJ15: CVCL_2678; SAS: CVCL_1675; SCC-9: CVCL_1685) used were maintained in DMEM/Nutrient Mixture F12 medium (Gibco; DMEM/F12) supplemented with 10% (v/v) heat-inactivated FBS (Gibco) and 100 IU penicillin/streptomycin (Gibco), as previously described (5). The ORL series of cell lines were established in Cancer Research Malaysia (15). All the cells were cultured in a humidified incubator containing 5% carbon dioxide at 37°C. The authenticity of the lines was confirmed by short tandem repeat profiling using a Promega PowerPlex 16 HS System, and the lines were routinely tested to ensure *Mycoplasma*-free status using MycoAlert PLUS Mycoplasma Detection Kit (Lonza Bioscience).

### Compound library

We curated a list of anticancer compounds, mainly comprising those clinically approved and in preclinical investigation, from the Genomics of Drug Sensitivity in Cancer library of compounds (16, 17). We also analyzed the sensitivity profile of OSCC lines included in the PRISM drug repurposing dataset (18) and included additional compounds that show selectivity and sensitivity (>3 sensitive OSCC lines; *n* = ∼39). Furthermore, we also derived a list of compounds targeting the OSCC essential genes using the PanDrugs tools (*n* = ∼30; refs. 18, 19). The compounds were sourced from commercial vendors and are listed in Supplementary Table S1.

### Drug screen

DMSO-solubilized compounds were stored at room temperature in low-humidity (<12% relative humidity) and low-oxygen (<2.5%) environments using storage pods (Roylan Developments). Water-soluble compounds were maintained at 4°C.

The seeding density was optimized before screening to ensure that each cell line was in the exponential growth phase at the end of the assay. Six seeding densities were tested via a twofold dilution step, and the cells were dispensed into 48 or 224 wells of a single 384- or 1,536-well assay plate, respectively, and incubated for 96 hours. The cell number was quantified using CellTiter-Glo 2.0 (Promega). The maximum density tested was 2,500 cells per well in the 384-well format and 1,250 cells per well in the 1,536-well format.

For the discovery screen, drugs were screened at seven concentrations spanning a 1,000-fold range with a half-log 3.16-fold dilution series. The cells were transferred to 1,536-well assay plates in 7.5 μL of DMEM/F12 using Multidrop Combi dispensers (Thermo Fisher Scientific). For the focused screen containing nine IAP inhibitors, the drugs were screened at 12 concentrations spanning a 2,048-fold range with a twofold dilution series.

The cells were transferred to 384-well assay plates in 40 μL of DMEM/F12. For both screens, 24 hours after seeding, assay plates were dosed with the test compounds using Echo 555 (Labcyte). The final DMSO concentration was 0.1%. The plates were incubated for a duration of drug treatment of 72 hours. CellTiter-Glo 2.0 (Promega) was added to measure cell viability (10 μL in 384-well plates and 2.5 μL in 1,536-well plates). The assay plates were incubated at room temperature for 10 minutes prior to quantification of luminescence using a paradigm plate reader (Molecular Devices). To estimate cell growth over the duration of drug treatment, an additional undrugged control plate was generated and cell viability was measured at the time of drug treatment. These plates are referred to as “day 1” plates, and the process was repeated each time a cell line was screened.

### Screen data processing and quality control

All screening plates contained negative control wells (untreated wells, *n* = 6/6; DMSO-treated wells, *n* = 62/126) and positive control wells (medium-only wells, *n* = 14/28; staurosporine-treated wells, *n* = 8/20; and MG132-treated wells, *n* = 8/20) distributed across 384/1,536 plates. These control wells were used to determine whether the individual plates met the defined quality control criteria. A maximum threshold of 0.18 was applied to the coefficient of variation of the DMSO-treated negative controls (CV = *σ*_*N*_/*μ*_*N*_, in which CV is the coefficient of variation, *σ*_*N*_ is the SD of the negative control, and *μ*_*N*_ is the mean of the negative control). Using the DMSO-treated negative control (NC1) and the two positive controls (PC1 and PC2), we determined the *Z*-factors (also known as *Z*′; *Z*-factor = 1 − 3 × (*σ*_*P*_ + *σ*_*N*_)/(|*μ*_*P*_ − *μ*_*N*_|), in which *σ*_*N*_ and *σ*_*P*_ are the SD of the negative and positive controls, respectively, and *μ*_*N*_ and *μ*_*P*_ are the mean of the negative and positive controls, respectively. *Z*-factors were calculated for all plates to indicate the sensitivity of the cell lines to the positive control (ratio of NC1:PC ≥ 4). In case of a cell line being insensitive to both positive control drugs, the *Z*-factors were calculated on the basis of blank wells instead. *Z*-factors were required to exceed a minimum threshold of 0.3 for individual plates and a mean of 0.4 across all plates within a screening set. Where a cell line was sensitive to both positive controls, it had to pass the *Z*-factor threshold for both positive controls. Plates that did not meet these requirements were excluded from this study.

### Curve fitting

Fluorescence sensitivity measurements for each plate in the drug screen were normalized to a cell viability scale using DMSO-treated negative controls (viability of 1) and positive controls (culture medium without cells; viability of 0). Two-parameter logistic dose–response curves were fitted to the drug treatments using a nonlinear mixed-effects model (20).

### Integration analysis of the drug screen and CRISPR screen

To identify drugs that can target genetic vulnerabilities of OSCC, we correlated the CRISPR score of essential genes with the IC_50_ of each compound. Considering a threshold IC_50_ of 1 µmol/L to be sensitive, we short-listed 72 “selective drugs,” for which 10% to 90% of the OSCC cell lines were sensitive (IC_50_ < 1 µmol/L). Among the 918 essential genes previously identified from the 21 OSCC lines, 388 were enriched in at least three OSCC lines (*n* ≥ 3). We then calculated the pairwise Pearson correlation coefficient (Pearson R) of the 72 drugs × 388 genes, which resulted in 27,936 drug–gene pairs, and their effect sizes (Cohen's d) between the dependent and nondependent groups. The Pearson correlation coefficient and *P* value (*cor.test*), as well as the effect sizes (“effsize” package), were calculated using RStudio (version 1.2.1335, RStudio, Inc., RRID: SCR_000432). Custom R code can be accessed via https://codeocean.com/capsule/7346043/tree/v1. Drug–gene pairs that had statistically significant Pearson correlations and effect sizes were further short-listed.



### Similarity analysis of IAP inhibitors

Pearson correlation analysis was used to evaluate the similarity between IAP inhibitors on the focused screen using the IC_50_ matrix of all nine IAP inhibitors for 21 OSCC lines.

### CRISPR/Cas9 knockout of target genes

Cas9-expressing OSCC cell lines were transduced with lentivirus carrying a gene-specific single-guide RNA (sgRNA) in pKLV2-U6gRNA5(BbsI)-PGKpuro2ABFP-W (RRID: Addgene_67991) as previously described (5). Two sgRNAs were used for each target gene. The first sgRNA was selected from Kosuke Yusa CRISPR Library v1 and coined with the abbreviation “1k.” The second sgRNA was either “2m” if chosen from the recommended MinLibCas9 library or “2b” if selected from the Broad Institute sgRNA design portal. A list of sgRNA sequences is provided in Supplementary Table S2.

### Apoptosis assays

On day 4 after transduction of sgRNA-containing lentiviruses, 30,000 cells were selected using puromycin, seeded into 24-well plates, and harvested for apoptosis assays after 72 hours.

To assess the effect of drug treatment, cells were seeded and treated with the indicated concentrations of AZD5582 for 72 hours. Upon harvesting, cells stained with propidium iodide and annexin V (BD Biosciences) were analyzed using an LSRFortessa X-20 Cell Analyzer (BD Biosciences) and FlowJo (version 10.5.3, BD Biosciences, RRID: SCR_008520), respectively (5).

### Cell viability assay

To determine the IC_50_ of AZD5582, cells were seeded at the optimized density in triplicate. The following day, various concentrations of AZD5582 were added to the cells, which were then incubated for 72 hours. The 3-(4,5-dimethylthiazol-2-yl)-2,5-diphenyltetrazolium bromide (MTT) was added to the cells, which were incubated for 4 hours at 37°C. After aspirating the MTT-containing medium, DMSO was used to dissolve the formazan crystals. Optical density at 570 nm was determined using a Synergy H1M Microplate Reader (BioTek Instruments).

### Colony formation assay

Cells were seeded into 24-well plates at an optimized cell density. To assess the impact of target gene knockout using sgRNA, posttransduced cells were seeded on day 4 and harvested 1 week later. For drug treatment, cells were seeded, the drug was added the day after seeding, and the cells were harvested 1 week later.

### Lysate preparation and Western blotting

For the baseline or changes in protein expression levels after gene knockout, lysates were prepared as previously described. To assess the impact of various concentrations of AZD5582 on expression level changes, cells were seeded in 100-mm^3^ dishes aiming at ∼20% to 30% confluency on the next day for drug treatment. Cells were harvested at the indicated time points. Lysates were collected, measured, and resolved using SDS-PAGE during Western blotting. A list of primary and secondary antibodies is shown in Supplementary Table S3. Uncropped images of the Western blots are shown in Supplementary Fig. S6.

### RNA sequencing of DMSO- or AZD5582-treated cells

AZD5582-sensitive and -resistant OSCC cells were seeded in 100 mm^3^ dishes and treated with 10 nmol/L AZD5582 or an equivalent concentration of DMSO as the control on the following day. Total RNA was extracted from a biologically duplicated set of samples from each cell line (control and 24-hour–treated samples). RNA sequencing (RNA-seq) was performed using an Illumina HiSeq 4000 system at the Wellcome Sanger Institute, with 35× to 60× transcriptomic coverage, and the data were processed using the iRAP pipeline, as previously described (bioRxiv 2014.005991). Briefly, paired-end transcriptome reads were quality-filtered and mapped to GRCh38 (Ensembl Build 98) using STAR v2.5.0c (21) with a standard set of parameters (https://github.com/cancerit/cgpRna). The resulting BAM files were processed to obtain per-gene read count data using HTSeq 0.7.2 (RRID: SCR_005514; ref. 22). Fragments per transcript per million were calculated and used for subsequent analyses.

### Differentially expressed gene analysis

For each cell line, the differentially expressed genes (DEG) between the AZD5582-treated and DMSO-treated controls were computed using the limma package (RRID: SCR_010943; Bioconductor, RRID: SCR_006442) based on duplicated data in the iRAP-processed log_2_(FPKM + 1) dataset, in which FPKM is fragments per transcript per million. Significant DEG hits were defined as those with a *P* value < 0.01 and log_2_ fold change >2. Heatmaps were plotted for better visualization using Morpheus software, developed by the Broad Institute (https://software.broadinstitute.org/morpheus).

### Gene set enrichment analysis

Gene set enrichment analysis (GSEA; RRID: SCR_003199) was performed using the GenePattern web tool (https://www.genepattern.org/; ref. 23) and the Broad Institute’s Molecular Signatures Database hallmark (comparing the baseline gene expression signatures of AZD5582-sensitive vs. AZD5582-resistant lines) or Kyoto Encyclopedia of Genes and Genomes (KEGG) gene sets (comparing the gene expression signatures of AZD5582-sensitive vs. AZD5582-resistant lines) as reference databases (24).

### Ethics approval

Animal ethics approval was obtained from the Universiti Kebangsaan Malaysia Animal Ethical Committee (CRM/2019/ANNIE CHAI/20-MAR./997-APR.-2019- DEC.-2023).

### Animal study

Five million ORL-207 cells were injected subcutaneously into both the flanks of specific pathogen-free–grade NOD/SCID mice (Nomura Siam International Co., Ltd, Thailand) at approximately 6 to 8 weeks of age. When the xenografts exceeded an average volume of 100 mm^3^, the mice were randomly assigned to the control or treatment arms and subjected to weekly intraperitoneal dosing of AZD5582 (10 mg/kg initial dose, followed by 3 mg/kg the subsequent weeks). Mice were monitored daily, and tumor volume and body weight were measured twice weekly. Tumor volumes were estimated using the formula 0.5 × length × width^2^. Mice were sacrificed after 3 weeks, and tumors were harvested, weighed, and processed to obtain protein lysates.

### ELISA

A total of 1.5e5 cells were seeded in a 24-well plate, and 24 hours later, cell culture supernatants at baseline and after 6 hours of treatment of AZD5582, 250 ng/mL lipopolysaccharide, or 10 ng/mL phorbol 12-myristate 13-acetate and ionomycin were collected. The levels of soluble TNF secreted into the cell culture supernatants were detected using RayBio Human TNF alpha ELISA Kit (#ELH-TNFa-1), following the manufacturer’s instruction. Quantification of TNF concentration was performed using the analysis template provided by the manufacturer, accessed from https://www.raybiotech.com/human-tnf-alpha-elisa-elh-tnfa.

### Statistical analyses

All statistical analyses were performed using an unpaired parametric two-tailed *t* test in GraphPad Prism (version 9.2.0; RRID: SCR_002798; GraphPad Software, Inc.), unless otherwise stated. Correlation analyses were performed by computing Pearson correlation coefficients using GraphPad Prism (RRID: SCR_002798).

### Data availability

Supplementary Dataset 1: JSON file containing drug response data for 339 compounds; Supplementary Dataset 2: RNA-seq–derived gene expression matrix. All the above datasets can be downloaded from figshare at https://figshare.com/s/68454e2fb832b526e7d8. The genomic and transcriptomic data of the OSCC cell lines used can be found in Cell Model Passports. RNA-seq BAM files of DMSO- and AZD5582-treated OSCC lines were deposited in the European Genome-phenome Archive (EGA50000000187).

Custom code used for integrative analyses of drug screen and CRISPR screen data can be accessed via Code Ocean at https://doi.org/10.24433/CO.3932688.v1.

## Results

### Integrative CRISPR-Cas9 and compound screening identifies AZD5582 as a potent inhibitor of OSCC

**Figure 1:**
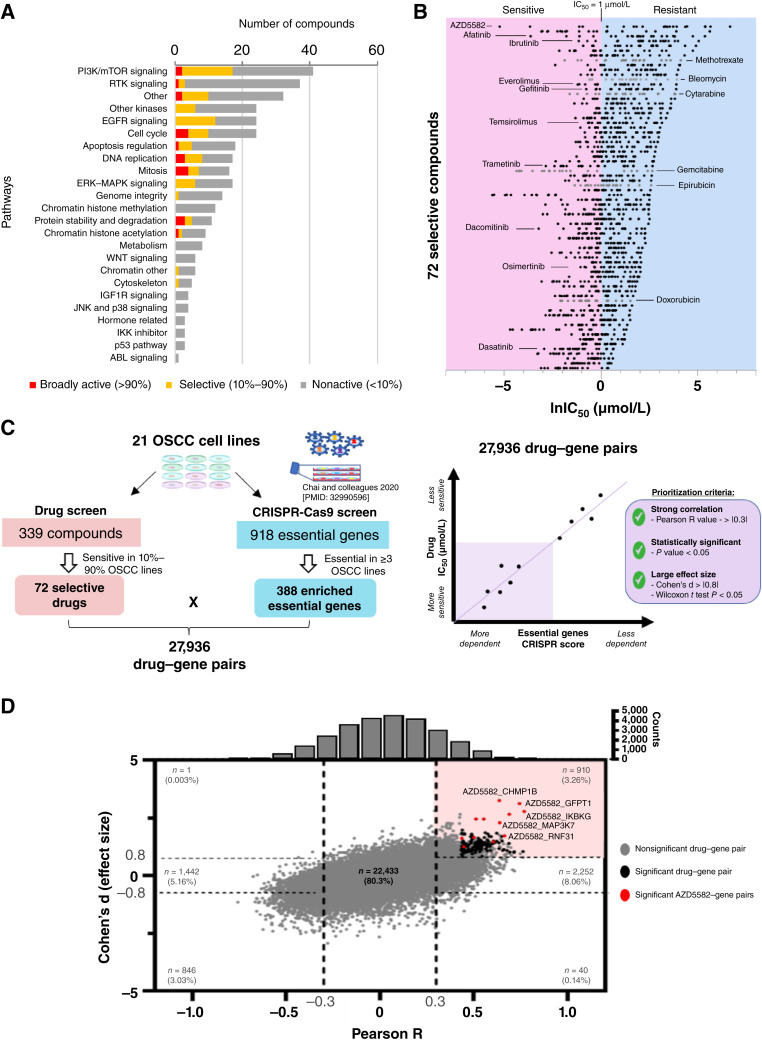
High-throughput compound screening identifies AZD5582 as a potent inhibitor that targets OSCC vulnerabilities. **A,** Barcharts showing pathway coverage of the 339 curated compounds screened on 21 OSCC cell lines. Using IC_50_ of <1 µmol/L as the cutoff for “sensitive” lines, we classified the compounds as broadly active (>90% of the cell lines being sensitive; red), selective (between 10% and 90% of the cell lines being sensitive; orange), and nonactive (<10% of the cell lines being sensitive; gray). **B,** Dot plots of the natural log of IC_50_ (lnIC_50_) for the 72 selective compounds (sorted by the largest range) across 21 OSCC cell lines. Sensitive cell lines are those with lnIC_50_ < 0 (IC_50_ < 1 µmol/L). **C,** Workflow and example of integration of CRISPR screen with drug screen data. The correlation between drug sensitivity and gene dependency is estimated using Pearson R, whereas the effect size is measured using Cohen's d effect size analysis. **D,** Plot of Cohen's d effect size vs. Pearson R for the 27,936 drug–gene pairs. The top right quadrant contains the most promising drug–gene pairs with a high level of correlation (Pearson R > 0.3) and huge effect size (Cohen's d > 0.8).

We have previously conducted a genome-wide CRISPR-Cas9 screen (5) in 21 OSCC cell lines to identify genetic vulnerabilities. To identify existing drugs that can be repurposed to target these vulnerabilities, we performed high-throughput compound screening on these 21 OSCC cell lines using a curated 339 compounds library (Supplementary Table S1). This dataset consists of FDA-approved cancer drugs and experimental drug candidates, including many present in the Genomics of Drug Sensitivity in Cancer database (16, 17), and compounds targeting OSCC essential genes, altogether covering 22 signaling pathways (Fig. 1A). Compounds were screened using a seven-point half-log dilution series spanning a 1,000-fold concentration range. A total of 7,119 dose–response curves were generated.

Using the IC_50_ of <1 µmol/L as the threshold for “sensitive” cell lines, we classified the compounds as broadly active (>90% of the cell lines were sensitive), potentially representing toxicity; selective (between 10% and 90% of the cell lines were sensitive, with a minimum of three sensitive lines); or inactive (<10% of the cell lines being sensitive; Fig. 1A; Supplementary Table S4). In total, there were 72 selected compounds, which included six chemotherapeutic drugs (Fig. 1B) and covered 15 pathways. Compounds targeting the PI3K/mTOR pathway dominate the library and had the highest proportion of selective compounds (15 of 42 compounds, 36%). EGFR inhibitors ranked second with 50% of the compounds being selective.

The success of targeted therapy has been attributed mainly to the preferential sensitivity of tumors with mutated oncogenes. In OSCC, *PIK3CA* is the most frequently mutated oncogene, and *PIK3CA* amplification and *PTEN* loss can also contribute to pathway activation. However, we did not observe an increase in PI3K inhibitor sensitivity in *PIK3CA*-mutated versus wild-type cells (Supplementary Fig. S1A). *PIK3CA*-dependent OSCC cell lines, as determined from CRISPR-Cas9 screens, consistently had lower IC_50_ values for PI3K inhibitors (Supplementary Fig. S1B), although this was not statistically significant.

**Figure 2:**
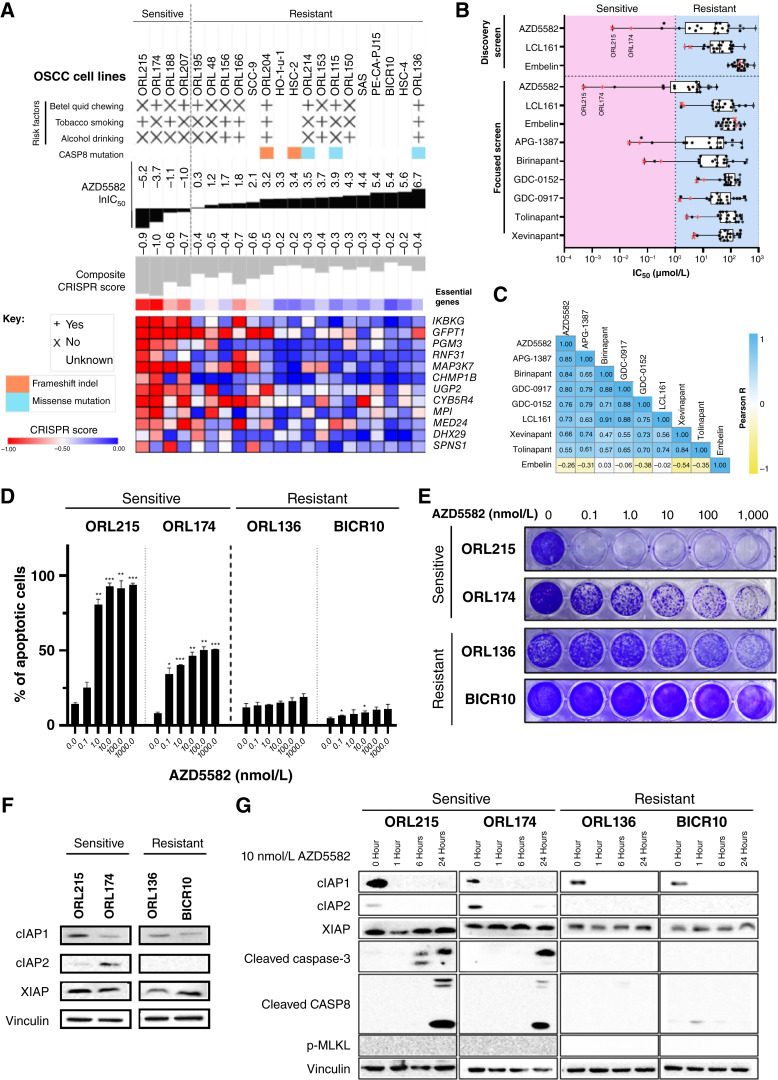
Validation of AZD5582 as a potent anticancer drug in a subset of OSCC via on-target degradation of cIAP1/2. **A,** Gene dependency and characteristics of the 21 OSCC cell lines according to the order of the most sensitive (lowest IC_50_) to least sensitive (highest IC_50_) response to AZD5582. **B,** Boxplots of IC_50_ values for three IAP inhibitors from the initial discovery screen and the focused screen with six additional IAP inhibitors. **C,** Similarity matrix of all nine IAP inhibitors tested in the focused screen, as estimated by the pairwise Pearson correlation R score using IC_50_ values. **D,** Dose-dependent effect of AZD5582 on OSCC cells as measured by apoptosis assay. Barcharts show the percentage of apoptotic cells after treatment with increasing concentrations of AZD5582. Error bars represent the mean ± SD of two biological repeats, each with technical triplicate. An unpaired Student *t* test was used with an untreated control (0 nmol/L AZD5582) as the comparator. *, *P* < 0.05; **, *P* < 0.01; ***, *P* < 0.005; ns, not significant. **E,** Effect of AZD5582 on colony formation in sensitive and resistant OSCC cell lines. **F,** Baseline expression of AZD5582 putative targets cIAP1, cIAP2, and XIAP. **G,** Time-dependent effect of 10 nmol/L AZD5582 demonstrating the on-target degradation of IAPs, as measured by Western blotting.

To identify drugs that can be used to target OSCC vulnerabilities and help delineate drug mechanisms of action, we systemically correlated the CRISPR essentiality gene score (CRISPR score) and drug sensitivity data (IC_50_ values), investigating 27,936 drug–gene pairs (Fig. 1C). There were 142 positively correlated drug–gene pairs (threshold applied: *P* value < 0.05, Pearson R > 0.3, and Cohen's d > 0.8; Fig. 1D; Supplementary Table S5). The highest correlated drug–gene pair was AZD5582 with *IKBKG* (Pearson R = 0.768 and *P* value = 4.76E−5). AZD5582 had 12 interacting genes (Fig. 2A), contributing to 8.4% of the significantly correlated drug–gene pairs.

### AZD5582 has potent activity in OSCC through on-target degradation of cIAP1/2

AZD5582 is a small-molecule inhibitor that targets the IAP family, the putative targets of which are cIAP1 (encoded by *BIRC2*), cIAP2 (encoded by *BIRC3*), and XIAP (encoded by *XIAP*). Four OSCC cell lines were sensitive to AZD5582, with IC_50_ values of 0.005 µmol/L (ORL-215), 0.025 µmol/L (ORL-174), 0.323 µmol/L (ORL-188), and 0.386 µmol/L (ORL-207). By contrast, the four most resistant OSCC cell lines had estimated IC_50_ values of >200 µmol/L. Notably, among the 72 selective compounds, AZD5582 had the largest range of IC_50_ values with a binomial drug sensitivity profile, suggesting strong drug selectivity. Given this high selectivity and robust correlation with fitness genes, we prioritized mechanistic investigation on AZD5582 to understand how this inhibitor can be used to treat OSCC.

*CASP8* plays a key role in apoptosis, and its mutation is enriched in Asian patients with OSCC (6). Notably, all *CASP8*-mutated OSCCs were resistant to AZD5582 (IC_50_ > 10 µmol/L; Fig. 2A). Three IAP inhibitors (AZD5582, LCL161, and embelin) were included in our initial discovery screen. To confirm AZD5582 sensitivity profiles, we independently tested six additional IAP inhibitors on 21 OSCC cell lines and used a wider concentration range (see “Materials and Methods” for details). We confirmed that AZD5582 is selectively potent in the same four OSCC lines, and when compared with all the other IAP inhibitors tested, AZD5582 had the lowest average IC_50_ (Fig. 2B). The activities of AZD5582 (Pearson R = 0.6184; *P* value = 0.0028) and LCL161 (Pearson R = 0.8906; *P* value < 0.0001) were consistent across the two screens (Supplementary Fig. S2A and S2B). Embelin, derived from a natural herb, showed little or no activity in either screen (Supplementary Fig. S2C). All other IAP inhibitors showed similar sensitivity profiles (Fig. 2C; Supplementary Fig. S2D). ORL-215 and ORL-174 were consistently the most sensitive lines for IAP inhibitors, including AZD5582. These data support the notion that a subset of OSCC cell lines is sensitive to IAP inhibitors in an on-target manner.

We selected the two most sensitive and resistant OSCC cell lines for confirmatory and mechanistic studies. AZD5582 resulted in a dose-dependent increase in apoptotic cells in ORL-215 and ORL-174 but not in resistant lines, ORL-136 and BICR10 (Fig. 2D). The apoptotic markers cleaved caspase 3 and CASP8 were induced only in the AZD5582-sensitive lines at 24 hours after treatment. Furthermore, we observed that compared with the resistant strains, the selective, potent subnanomolar AZD5582 inhibited colony formation of ORL-215 and ORL-174 (Fig. 2E). The most sensitive line ORL-215 showed the highest cIAP1 protein expression, whereas ORL-174 showed the highest cIAP2 expression (Fig. 2F). cIAP2 was detected in the sensitive lines ORL-174 and ORL-207 but not in the resistant lines (Supplementary Fig. S2E). Moreover, there was no difference in XIAP expression between cell lines. Treatment of OSCC cell lines with 10 nmol/L AZD5582 led to complete degradation of cIAP1 and cIAP2 as early as 1 hour after treatment, regardless of whether the cell line was sensitive or resistant (Fig. 2G).

Taken together, we validated findings from the discovery drug screen that IAP inhibitors, especially AZD5582, are selectively potent in the subset of OSCCs via on-target degradation of cIAP1 and cIAP2.

### AZD5582 sensitivity is correlated with dependency on essential genes in the NF-κB pathway

**Figure 3:**
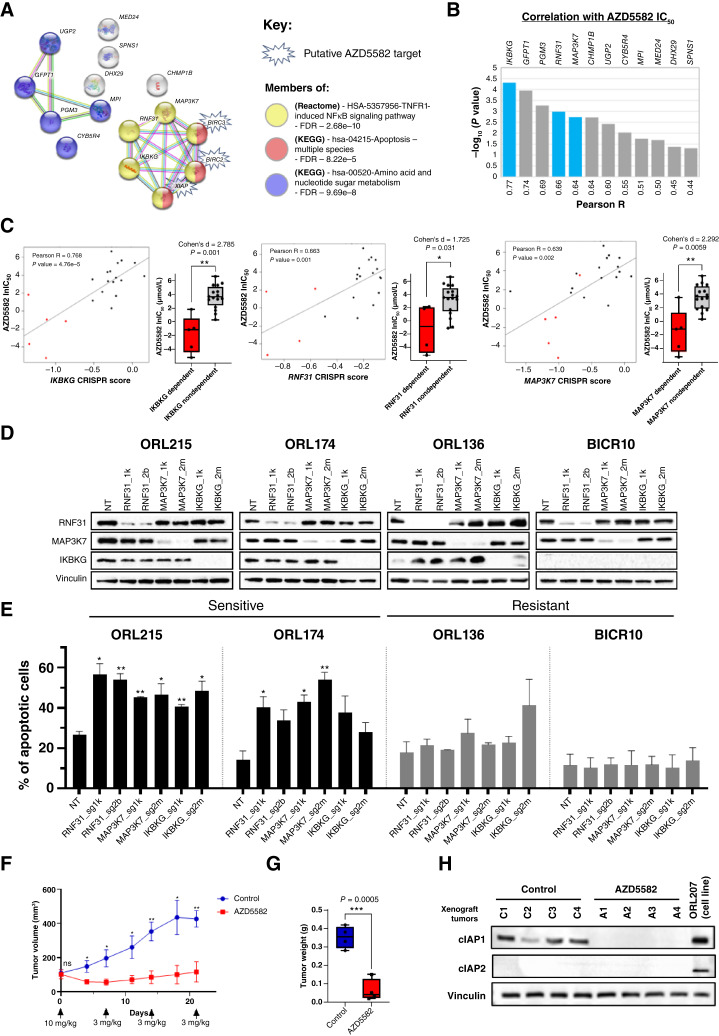
AZD5582 sensitivity is correlated with dependency on fitness genes from the NF-κB pathway. **A,** The STRING protein–protein interaction network of AZD5582 targets (BIRC2, BIRC3, and XIAP) and 12 fitness genes correlated with drug sensitivity. Default parameters were applied, and the relevant significantly enriched pathways are highlighted. **B,** Twelve correlated fitness genes ranked by the significant level of their correlation with AZD5582. Blue bars highlight the three fitness genes from the NF-κB pathway that are directly connected to AZD5582 putative targets. **C,** Correlation plots of AZD5582 lnIC_50_ (*y*-axis) and CRISPR score of the three NF-κB pathway fitness genes (*IKBKG*, *RNF31*, and *MAP3K7*) and boxplots of the dependent and nondependent OSCC cell lines. Red, sensitive lines. **D,** Western blots showing the effective knockout of fitness genes in sensitive and resistant OSCC cell lines. **E,** Bar charts showing the percentage of apoptotic cells induced by depletion of the fitness genes. Error bars show the mean ± SD of two biological repeats, each with technical triplicate. An unpaired Student *t* test was used, with nontargeting (NT) serving as a comparator. *, *P* < 0.05; **, *P* < 0.01; ***, *P* < 0.005. **F,** The tumor growth curve of ORL-207 xenografts demonstrated the *in vivo* antitumor efficacy of AZD5582. **G,** Boxplot showing the weights of tumor xenografts harvested from the control or AZD5582-treated mice. **H,** Western blot analysis of ORL-207 xenografts confirm the on-target degradation of cIAP1 by AZD5582. Control group tumors (C1, C2, C3, and C4), AZD5582 group tumors (A1, A2, A3, and A4), and ORL-207 cell line lysates were used as the positive controls. lnIC_50_, natural log of IC_50_.

Among the 12 genes correlated with AZD5582 sensitivity, three genes (*IKBKG*, *RNF31*, and *MAP3K7*) encoded proteins connected to IAP proteins encoded by *BIRC2* (cIAP1), *BIRC3* (cIAP2), or *XIAP* in the STRING protein‒protein interaction network (Fig. 3A and B). Based on functional enrichment analyses using the Reactome and KEGG databases, these three genes, together with the drug targets, belong to the “TNFR1-induced NF-κB signaling pathway” (Reactome database; FDR = 2.68e-10). Five other genes that correlated with AZD5582 sensitivity (*UGP2*, *GFPT1*, *PGM3*, *MPI*, and *CYB5R4*) were associated with “amino acid and nucleotide sugar metabolism” (KEGG database; FDR: 9.69e-10). The three NF-κB pathway genes were among the genes most strongly correlated with AZD5582 sensitivity, with large statistically significant effect sizes (Cohen's d > 0.8; *P* value < 0.05; Fig. 3C; Supplementary Fig. S3A–S3I). To confirm the role of the NF-κB pathway in modulating sensitivity toward AZD5582, we showed that the baseline NF-κB activity was higher in the sensitive line than in the resistant line and that NF-κB activity showed a dose-dependent decrease upon treatment with AZD5582, exclusively in the sensitive line (Supplementary Fig. S3J). In line with the observation that AZD5582-sensitive lines have more activated baseline NF-κB activity, we also showed that they have lower baseline IκBa levels, although having higher phosphorylated p65 (Supplementary Fig. S3K). We also noticed that in the most sensitive line ORL-207, AZD5582 resulted in an upregulation of the IκBa level, indicating that the NF-κB pathway is being deactivated and hence less degradation of IκBa.

Owing to the direct connection between the three NF-κB pathway genes and IAPs, we confirmed their fitness effect on OSCC. Upon sgRNA-mediated knockout of *RNF31*, *MAP3K7*, and *IKBKG*, there was an increase in the percentage of apoptotic cells in ORL-215 and ORL-174 (except for *IKBKG*, which did not reach statistical significance), whereas the AZD5582-resistant lines did not show dependency on these genes for survival (Fig. 3D and E). Depletion of these genes also had a greater inhibitory effect on colony formation ability among the AZD5582-sensitive lines (Supplementary Fig. S3L). Taken together, these data confirm that the effects of AZD5582 on OSCC cell lines are dependent on the NF-κB pathway.

### AZD5582 suppresses OSCC tumor xenografts

To assess the efficacy of AZD5582 in controlling OSCC tumor growth, we subcutaneously inoculated all four AZD5582-sensitive cell lines into NOD/SCID mice. Only ORL-207 formed robust tumors and was selected for further investigation. Weekly treatment of established (100 mm^3^) tumors with AZD5582 resulted in significant tumor growth inhibition when compared with that of the controls and a significant reduction in tumor weight after 21 days of treatment (Fig. 3F and G). AZD5582 led to complete and durable degradation of cIAP1 in tumor cell lysates (Fig. 3H). For reasons that remain unclear, cIAP2 was not detected in xenografts but was highly expressed *in vitro*. Mice treated with AZD5582 exhibited some weight loss 3 days after injection but gradually regained weight before the next time point (Supplementary Fig. S3M). The results show that AZD5582 was highly efficacious for OSCC antitumor control *in vivo*.

### AZD5582-induced apoptosis is associated with NF-κB pathway inhibition

To determine the mechanism of AZD5582 sensitivity, we used GSEA on the 50 hallmark gene sets, comparing baseline gene expression between the four sensitive OSCCs and 17 resistant OSCCs. Consistent with the finding that AZD5582 induces apoptosis in NF-κB pathway–dependent OSCC cells, the hallmark “apoptosis” (normalized enrichment score = 1.468; nominal *P* value = 0.005) and “TNF signaling via NF-κB” (normalized enrichment score = 1.200; nominal *P* value = 0.043) were significantly enriched in the AZD5582-sensitive OSCC (Supplementary Fig. S4A and S4B).

**Figure 4:**
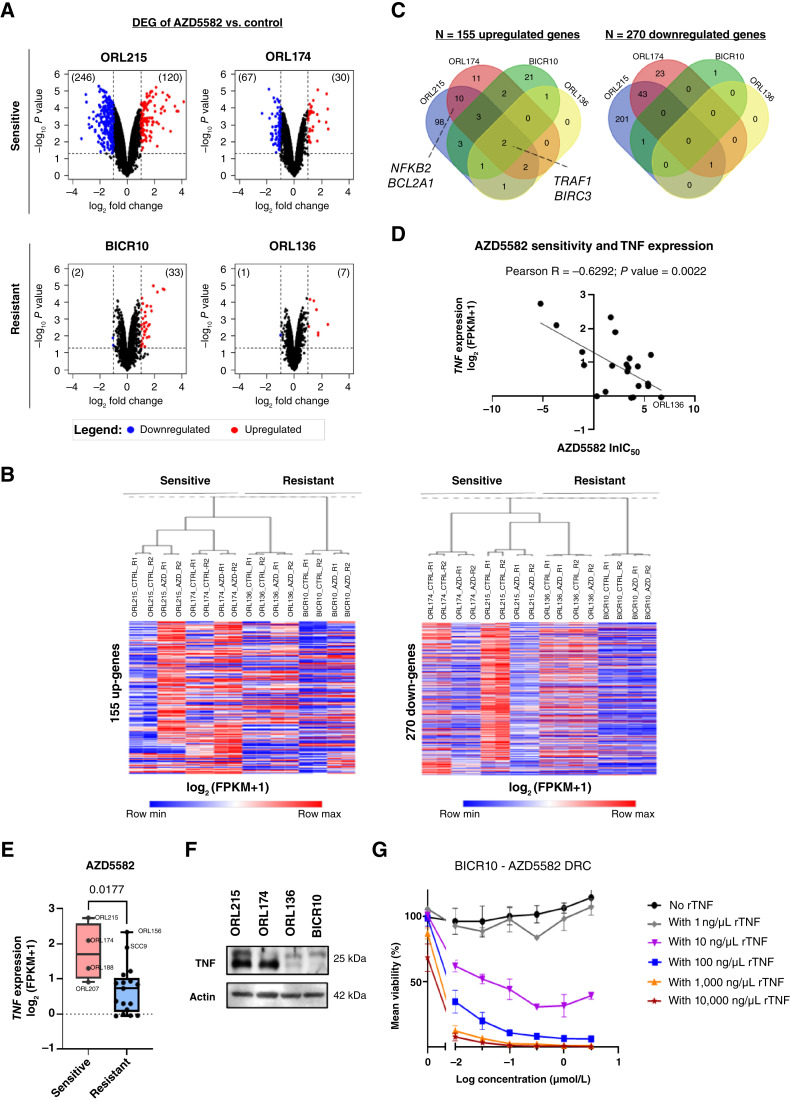
Gene expression analysis reveals that AZD5582-induced apoptosis is associated with inhibition of the NF-κB pathway and high TNF levels. **A,** Volcano plots showing the overall transcriptomic changes induced by AZD5582 treatment in sensitive and resistant OSCC cell lines. **B,** Heatmap of differentially up- or downregulated genes from the RNA-seq analysis of DMSO control and AZD5582-induced transcriptomic changes in sensitive and resistant OSCC cell lines. **C,** Venn diagram showing the number of differentially up- or downregulated genes upon AZD5582 treatment. **D,** Correlation plot of TNF expression from RNA-seq data of 21 OSCC cell lines with the natural log of IC_50_ (lnIC_50_). of AZD5582. **E,** Boxplot showing the difference in TNF expression between AZD5582-sensitive and AZD5582-resistant OSCC. **F,** Western blot showing the TNF protein level in AZD5582-sensitive and AZD5582-resistant OSCC. **G,** Dose–response curves (DRC) of BICR10 toward AZD5582 upon sensitization to increasing concentrations of recombinant TNF (rTNF).

To further delineate the mechanism of AZD5582 sensitivity, we performed RNA-seq and compared the gene expression for control (“CTRL”) and AZD5582-treated (“AZD”) OSCC, for the two sensitive lines (ORL-215 and ORL-174) and the two resistant lines (ORL-136 and BICR10). Technical duplicates for each condition showed a high concordance (Supplementary Fig. S4C). After 24 hours of treatment with as low as 10 nmol/L AZD5582, greater transcriptomic changes were induced in the sensitive lines (Fig. 4A; Supplementary Fig. S4D). A list of DEGs (*P* value threshold < 0.01 and log_2_ fold change > 2) is shown in Supplementary Table S6. The samples were clustered primarily by cell line and then by AZD5582 sensitivity, with BICR10 clustering separately from the other cell lines (Fig. 4B). Notably, *TRAF1* and *BIRC3* were upregulated independently of AZD5582 sensitivity, whereas *NFKB2* and *BCL2A1* were among the upregulated genes in the two sensitive lines (Fig. 4C; Supplementary Fig. S4E and S4F). The apoptosis pathway was among the significantly enriched KEGG pathways compared with that in AZD5582-treated resistant lines (Supplementary Fig. S4G). Given the enrichment of *TNF* genes according to our GSEA, we next examined the role of TNF in mediating AZD5582 sensitivity. Notably, *TNF* expression was correlated with increased AZD5582 sensitivity in OSCC cell lines (Pearson R = −0.6292; *P* value = 0.0022; Fig. 4D), and *TNF* expression was higher in AZD5582-sensitive lines than in AZD5582-resistant lines (*P* value = 0.0177; Fig. 4E). In addition, membrane-bound TNF protein was detected in the sensitive lines ORL-215 and ORL-174, whereas there was negligible expression in the resistant lines BICR10 and ORL-136 (Fig. 4F; Supplementary Fig. S4H). We also used the ELISA to detect the baseline level of soluble TNF secreted into the conditioned media. We confirmed that the secretion of TNF was also higher among the AZD5582-sensitive lines, at baseline and when stimulated by AZD5582 and other stimulants such as lipopolysaccharide and phorbol 12-myristate 13-acetate + ionomycin (Supplementary Fig. S4I). To directly ascertain whether AZD5582 sensitivity can be modulated by TNF, we treated the *CASP8* wild-type resistant line BICR10 with increasing concentrations of recombinant TNF. Recombinant TNF sensitized OSCC cells to AZD5582 in a dose-dependent manner (Fig. 4G). Conversely, the depletion of endogenous TNF desensitized ORL-174 to AZD5582 (Supplementary Fig. S4J). In summary, these data indicate that high TNF renders OSCC cells sensitive to AZD5582-induced cell death and suggest that TNF expression could be used as a biomarker of AZD5582 sensitivity.

### AZD5582 cytotoxicity can be mediated by CASP8-dependent apoptosis or CASP8-independent necroptosis

**Figure 5:**
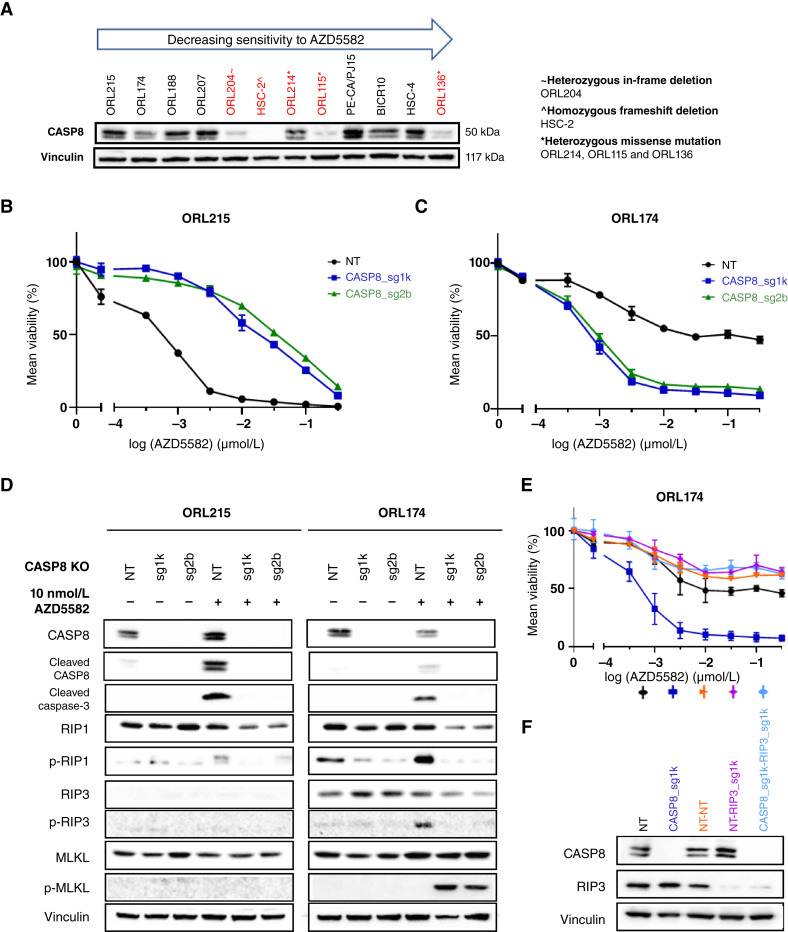
AZD5582 acts on OSCC predominantly via triggering CASP8-dependent apoptosis. **A,** Western blot showing CASP8 protein expression in wild-type and mutant OSCC (red). **B,** Dose–response curve on the effect of CASP8 knockout (KO) on AZD5582 sensitivity in ORL-215. **C,** Dose–response curve on the effect of CASP8 KO on AZD5582 sensitivity in ORL-174. **D,** Western blot showing the levels of apoptotic and necroptotic markers in CASP8 KO cells. **E,** Dose–response curve toward AZD5582 upon CASP8 KO and/or RIP3 KO, confirming the involvement of RIP3-dependent necroptosis in mediating the effect of AZD5582. **F,** Western blot confirming CASP8 and/or RIP3 KO in ORL-174.

*CASP8* is a commonly mutated gene in OSCC, and its prevalence is higher in Asian patients with OSCC (6). To investigate how *CASP8* mutation and protein levels are related to AZD5582 sensitivity, we first confirmed that all four AZD5582-sensitive lines expressed CASP8 protein. CASP8 was not detected in HSC-2 cells, which had a homozygous frameshift deletion; however, for cell lines with heterozygous mutations, CASP8 expression levels were typically low (Fig. 5A). To directly evaluate the role of CASP8, we generated *CASP8*-knockout isogenic versions of two sensitive lines (ORL-215 and ORL-174) and measured their AZD5582 sensitivity. Surprisingly, the opposite trends were observed. Upon loss of *CASP8*, ORL-215 cells became less sensitive to AZD5582 treatment, which was consistent with the finding that AZD5582 induced CASP8-mediated apoptosis (Fig. 5B); however, for ORL-174, AZD5582 sensitivity increased upon *CASP8* knockout (Fig. 5C).

In addition to mediating apoptosis, CASP8 also functions to inhibit necroptosis, another form of cell death (25). We hypothesized that upon deletion of *CASP8* in ORL-174, cells could be undergoing necroptosis. We first examined the expression of necroptotic pathway components RIP1, RIP3, and MLKL in *CASP8* isogenic lines (Fig. 5D). We observed that ORL-174 cells, but not ORL-215 cells, expressed RIP3. Furthermore, in the absence of *CASP8*, AZD5582 induced activation of the pro-necroptotic protein MLKL. Similarly, in another sensitive line (ORL-207) that also expressed RIP3, we observe a switch from apoptotic to necroptotic cell death when treated with AZD5582 (Supplementary Fig. S5A). The increase in p-MLKL levels in ORL-174 and ORL-207 cells with *CASP8* knockout is time dependent subsequent to AZD5582 treatment (Supplementary Fig. S5B). We also noted that the switch to necroptosis is not only unique to AZD5582 but also seen when the CASP8–knocked-out cells were treated with xevinapant, an IAP inhibitor undergoing clinical trial testing (Supplementary Fig. S5C). Notably, to directly test whether cells underwent RIP3-mediated necrosis in the absence of *CASP8*, we knocked out *CASP8* and *RIP3* individually and together in ORL-174 cells and evaluated AZD5582 sensitivity (Fig. 5E and F). Strikingly, the loss of RIP3 alone had no effect on AZD5582 sensitivity but completely reversed the increase in AZD5582 sensitivity in CASP8-deficient cells. Taken together, these results support a role for RIP3-dependent necroptosis in mediating AZD5582 sensitivity in the setting of CASP8-deficient OSCC, consistent with the findings of previous reports on another IAP inhibitor, birinapant (12).

**Figure 6:**
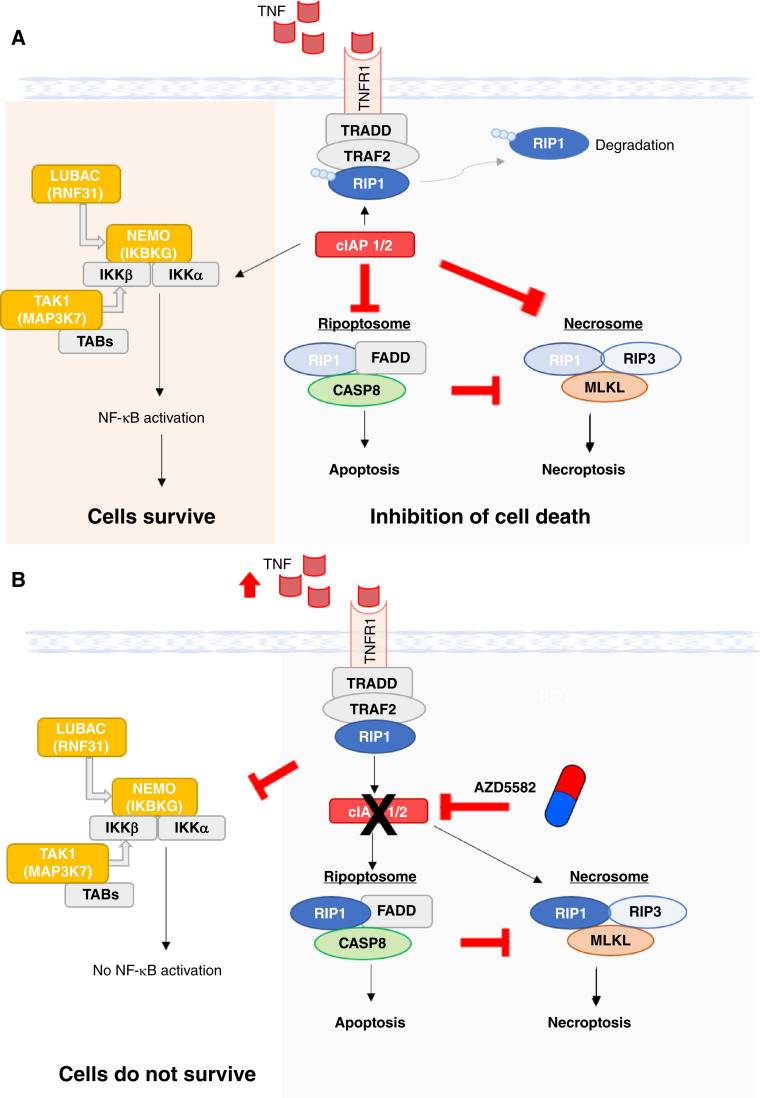
Proposed modes of mechanism of action of AZD5582 in OSCC. **A,** Upon activation by external death stimuli such as TNF, cIAP1/2 in OSCC trigger ubiquitination of RIP1, disabling its binding with FADD and CASP8 in the formation of ripoptosome complex, thereby preventing apoptosis. Likewise, necrosome formation is also inhibited. By contrast, cIAP1/2 promotes the ubiquitination cascade of LUBAC and IKK complexes, activating the NF-κB canonical signaling pathway, promoting cell survival. **B,** In the presence of high levels of TNF, AZD5582 degrades cIAP1/2, thereby preventing it from ubiquitinating RIP1. RIP1 forms the ripoptosome complex with FADD and CASP8, leading to cell death via apoptosis. Necroptosis is inhibited in the presence of CASP8, but on the contrary, if functional CASP8 is absent, the RIP1–RIP3–MLKL complex may lead to cell death via necroptosis. On the other hand, the NF-κB canonical signaling pathway is inhibited in the absence of cIAP1/2, impairing OSCC cell survival. TAB - TGF-Beta Activated Kinase 1 (MAP3K7) Binding Protein.

In conclusion, we propose a model in which a subset of OSCC cells with elevated TNF levels is dependent on cIAPs to induce the degradation of RIP1, thereby blocking the formation of the ripoptosome complex and prohibiting CASP8-dependent apoptosis. cIAPs can also activate the canonical NF-κB pathway, which includes the essential OSCC genes *RNF31*, *IKBKG*, and *MAP3K7*, resulting in sustained cell viability (Fig. 6A). Hence, treatment of NF-κB pathway–addicted OSCCs with AZD5582 leads to the degradation of cIAPs, triggering a cascade of events, including reduced survival signaling through the NF-κB pathway and stabilization of the ripoptosome complex, enabling CASP8-dependent apoptosis. When CASP8 is lost, OSCC cells with functional necroptosis machinery (RIP3 expressing) are vulnerable to AZD5582 treatment, during which cells die via the necroptotic pathway (Fig. 6B).

## Discussion

Genome-wide CRISPR-Cas9 screens have enabled the discovery of cancer vulnerabilities, some of which can potentially be targeted by repurposing existing drugs (26–29). In this study, we screened 339 clinically relevant compounds on 21 OSCC cell lines and integrated these data with CRISPR/Cas9 and genomic and transcriptomic data to generate a comprehensive resource for mechanism-guided drug repurposing for OSCC. We identified 142 positively correlated selective drug–gene pairs, indicating that patients with OSCC with specific vulnerabilities confer preferential sensitivity to the correlated compound. To the best of our knowledge, this is the first study to describe the outcomes of integrating CRISPR and drug screens to identify potential drug targets for OSCC. This strategy enables elucidation of the mechanism of action of drugs and facilitates the discovery of molecular markers that underpin drug sensitivity (1).

The most selective compound identified was AZD5582. Interestingly, all four AZD5582-sensitive Asian OSCC cell lines were associated with either betel quid chewing or tobacco smoking. Furthermore, three of the significantly correlated fitness genes belong to the NF-κB signaling pathway, which we previously showed is an enriched pathway of fitness genes among Asian patients with OSCC with betel quid chewing habit (5).

AZD5582 is a small dimeric molecule that mimics the endogenous inhibitor of IAPs, the second mitochondria-derived activator of caspase (SMAC; ref. 30). Overexpression of IAPs has been reported in OSCC, and targeting IAPs to induce apoptosis has been demonstrated to be a promising strategy for treating OSCC (7, 8). Interestingly, only the two betel quid–associated sensitive lines, ORL-174 and ORL-207, expressed high levels of cIAP2, which was not found to be expressed in the other lines. Regardless of the sensitivity toward AZD5582, cIAP1 and cIAP2 were effectively degraded. However, no changes in XIAP expression were observed, consistent with the reported consequences of SMAC coexpression in HeLa cells (31). In the context of OSCC, the cytotoxic effect of AZD5582 is likely attributable to the degradation of cIAP1/2, which mediates CASP8-dependent apoptosis but is less likely due to XIAP-mediated inhibition of the apoptosis initiator caspase-9, unlike what has been reported for other SMAC mimetics (32, 33).

Both TNF levels and dependency on NF-κB pathway genes are associated with AZD5582 sensitivity. The NF-κB pathway genes *RNF31* (HOIP), *IKBKG* (NEMO), and *MAP3K7* (TAK1) have all been separately indicated as potential therapeutic targets for various tumors (34–36). *RNF31* encodes HOIP, an atypical ubiquitin ligase that is part of the linear ubiquitin chain assembly complex (LUBAC), facilitating the activation of the IKK complex by the IKKβ kinase TAK1 (encoded by the *MAP3K7* gene; ref. 37). The IKK complex comprises two kinases, IKKα and IKKβ, and a regulatory subunit, IKKγ (encoded by the *IKBKG* gene). This subunit, also known as NEMO, is ubiquitinated by the LUBAC. Upon binding of TNF to the TNF receptor (*TNFR1*), a death complex comprising TRADD, TRAF2, and RIP1 is formed, and the cIAP-mediated polyubiquitination of RIP1 serves as a recruitment platform for the LUBAC and IKK complexes, eventually leading to the activation of canonical NF-κB signaling to induce prosurvival transcriptomic changes (38). Given the concerted role of these three fitness genes in transduction of the TNFR1-induced NF-κB pathway and cross-talk with cIAP-mediated death signaling pathways, we postulate that other cancer types dependent on NF-κB pathway genes are also likely to respond to IAP inhibitors.

*CASP8* is one of the top 10 mutated genes in OSCC, and its prevalence seems to be greater among Asian patients with OSCC (6). We demonstrated that cytotoxicity of AZD5582 is mainly mediated by *CASP8*-dependent apoptosis. Additionally, we provide evidence that AZD5582 can promote cell death through necroptosis when CASP8 is lost and necroptosis signaling is intact. Necroptotic cell death is more immunogenic than nonapoptotic cell death (39) and may favor responsiveness toward immunotherapy (40). However, the apoptosis–necroptosis switch could not be evaluated *in vivo* because of the overwhelming tumor suppression effect of AZD5582. Future investigations are warranted to determine the synthetic lethality relationship between *CASP8* loss and susceptibility to IAP inhibitor–induced necroptosis, which could potentially substantiate the response of CASP8-deficient OSCC cells toward radiotherapy, as seen in the case of birinapant (12) and immunotherapy.

AZD5582 induces OSCC cell death and suppresses tumor xenograft growth *in vivo*, but treatment-induced weight loss in mice suggests that dose and scheduling optimization may be required. Furthermore, the development of an AZD5582-like drug for human use, which is being explored for the treatment of human immunodeficiency virus as a latency-reversing agent, holds promise for clinical applications (41, 42). Our study could also be informative for other IAP antagonists such as birinapant, xevinapant, tolinapant (ASTX660), and APG-1387 (8, 43), which are being investigated in patients with HNC in phase II and III clinical trials (44). Our results indicate that tumors with high TNF levels and NF-κB signaling are the most responsive to IAP inhibitors. Importantly, although AZD5582 was the most potent IAP inhibitor, xevinapant had similar sensitivity profiles in OSCC cell lines. In particular, xevinapant is the most clinically advanced IAP inhibitor, with promising antitumor efficacy seen in unresected, locally advanced HNC (LA-HNC) in a phase II trial, when used in combination with CRT (ref. 8). This has led to two phase III trials for unresected LA-HNC with CRT (TrilynX) and cisplatin-ineligible resected LA-HNC with radiotherapy (XRay Vision; refs. 8, 43). Unfortunately, in a recent press release, the trials have been terminated as they are unlikely to meet the primary objective of prolonging event-free survival (45). Although in-depth review of the data is ongoing, our observation in this study is consistent and that only a subset of OSCC is responsive to AZD5582. It will be interesting to investigate if biomarkers of high TNF and* CASP8* mutational status correlate with the treatment outcome seen in the patients from both trials. It is likely that a biomarker-guided selection of patients will be needed for consideration of future trial design, of which our study provides useful insights on potential biomarkers of inclusion and exclusion.

The strength of our study lies in our systematic approach of integrating data from genetic and pharmacologic screens, offering a mechanistically driven approach to not only just discover active anticancer drug for repurposing but also enable insights into mechanisms underlying responses to these drugs. Besides, our study comprehensively studied molecular markers and the genomic context underpinning AZD5582 sensitivity, unveiling the complex prerequisite context for an effective antitumor effect. However, one of the inherent limitations of CRISPR screens is that they suffer from false negatives wherein genes with paralogs or isoforms with redundant functions may be missed. In this case, the IAPs which are direct targets of AZD5582 were missed out as they had redundant or compensatory functions that masked the effect of single-gene knockout. Our approach of looking at indirect targets (the NF-κB pathway genes) that interact with the direct target (IAPs) in a pathway-level approach enabled us to identify a highly selective drug like AZD5582. Our findings on the biomarkers of sensitivity and resistance for AZD5582 also require further validation using clinical specimens. Although we demonstrate that high TNF and strong dependency on NF-κB pathway are indications of response to AZD5582, OSCCs can be sensitized to the drug when given increasing doses of recombinant TNF. Given that the majority of patients with OSCC will be treated with radiotherapy, which is known to induce *TNF* and *TNFR1* expressions (46), tumors with intrinsically low TNF could potentially be sensitized to the IAP inhibitor. The detailed results from the recent phase III trials of xevinapant in combination with CRT are highly anticipated, and analyses of the biomarkers underpinning clinical responses will warrant further investigation to guide future clinical trial design.

## Conclusion

We have demonstrated IAP inhibitor sensitivity in OSCC *in vitro* and *in vivo*, and this is associated with TNF and NF-κB signaling. We elucidated two mechanisms of AZD5582-induced cell death (apoptosis or necroptosis), depending on the mutational status of *CASP8* or the functional necroptotic machinery, as indicated by RIP3 expression. Our findings could inform the clinical development of IAP inhibitors, particularly for OSCC, and more broadly demonstrate the utility of integrative pharmacologic and genetic screens to inform mechanistically guided opportunities for drug repurposing.

## Supplementary Material

Supplementary Figure 1 *PIK3CA*-dependent OSCC showed higher sensitivity towards PI3K inhibitors.

Supplementary Figure 2 A subset of OSCCs is vulnerable to AZD5582 and other IAP inhibitors.

Supplementary Figure 3 Correlation of AZD5582 sensitivity with OSCC essential genes.

Supplementary Figure 4 Investigation of gene expression changes associated with AZD5582.

Supplementary Figure 5 Necroptosis is activated in the absent of CASP8 upon AZD5582 treatment as shown by induction of p-MLKL.

Supplementary Figure 6 All uncropped western blot images

Supplementary Table 1 List of compounds screened, their putative targets and supplier info.

Supplementary Table 2 List of sgRNAs and their sequences

Supplementary Table 3 List of antibodies

Supplementary Table 4 ln IC50 data of 339 compounds in 21 OSCC cell lines

Supplementary Table 5 List of 142 significant positively correlated drug-gene pairs

Supplementary Table 6 Differentially expressed genes (DEGs) in AZD5582-treated cells compared to control
