# Supplementary material for: High TNF and NF-κB Pathway Dependency Are Associated with AZD5582 Sensitivity in OSCC via CASP8-Dependent Apoptosis
Source: Cancer Res Commun. 2024 Nov 11;4(11):2919–32. doi: 10.1158/2767-9764.CRC-24-0136 (PMC11551840; doi:10.1158/2767-9764.CRC-24-0136)
Supplement: Supplementary Figure 1 — PIK3CA-dependent OSCC showed higher sensitivity towards PI3K inhibitors. [file crc-24-0136_supplementary_figure_1_suppsf1.pdf]

Supplementary Figure 1

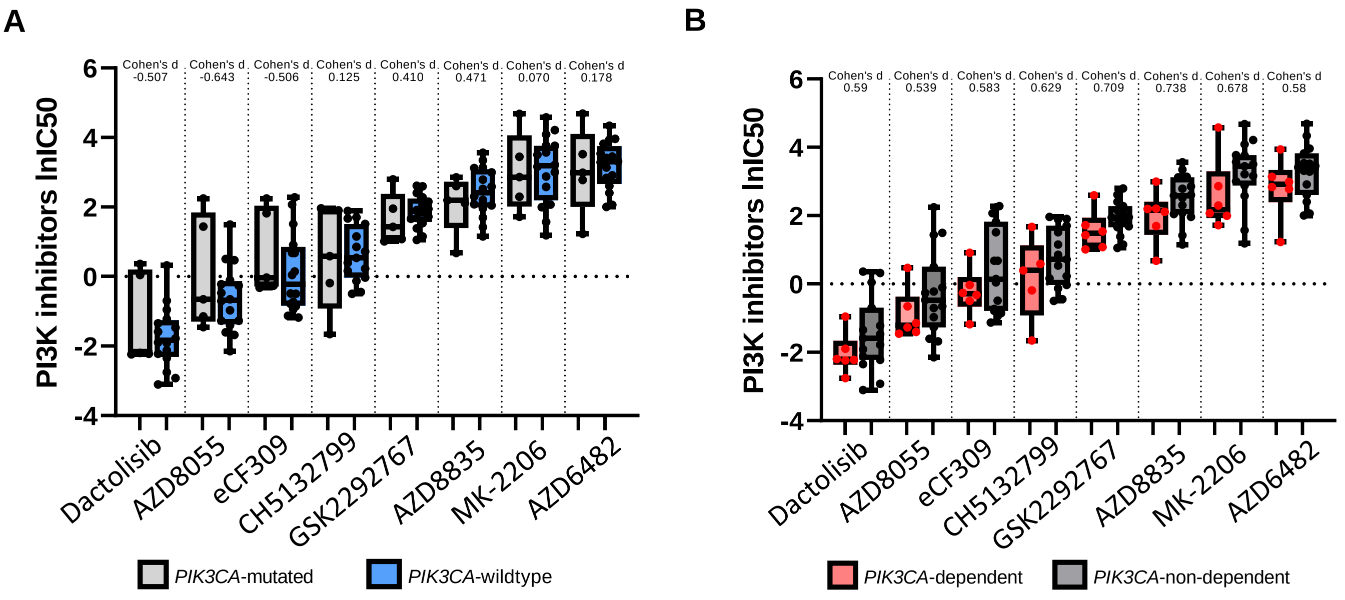

Supplementary Figure 1 – *PIK3CA*-dependent OSCC showed higher sensitivity towards PI3K inhibitors.

(A) OSCC cell lines that are dependent on *PIK3CA* showed lower average IC50 towards selective PI3K inhibitors. Only those with medium effect size between the two groups were shown. None are statistically significant (p-value > 0.05).

(B) *PIK3CA* mutation is not associated with higher sensitivity towards PI3K inhibitors. Effect sizes are either negligible or small. None are statistically significant (p-value > 0.05).
