# Supplementary material for: High TNF and NF-κB Pathway Dependency Are Associated with AZD5582 Sensitivity in OSCC via CASP8-Dependent Apoptosis
Source: Cancer Res Commun. 2024 Nov 11;4(11):2919–32. doi: 10.1158/2767-9764.CRC-24-0136 (PMC11551840; doi:10.1158/2767-9764.CRC-24-0136)
Supplement: Supplementary Figure 2 — A subset of OSCCs is vulnerable to AZD5582 and other IAP inhibitors. [file crc-24-0136_supplementary_figure_2_suppsf2.pdf]

Supplementary Figure 2

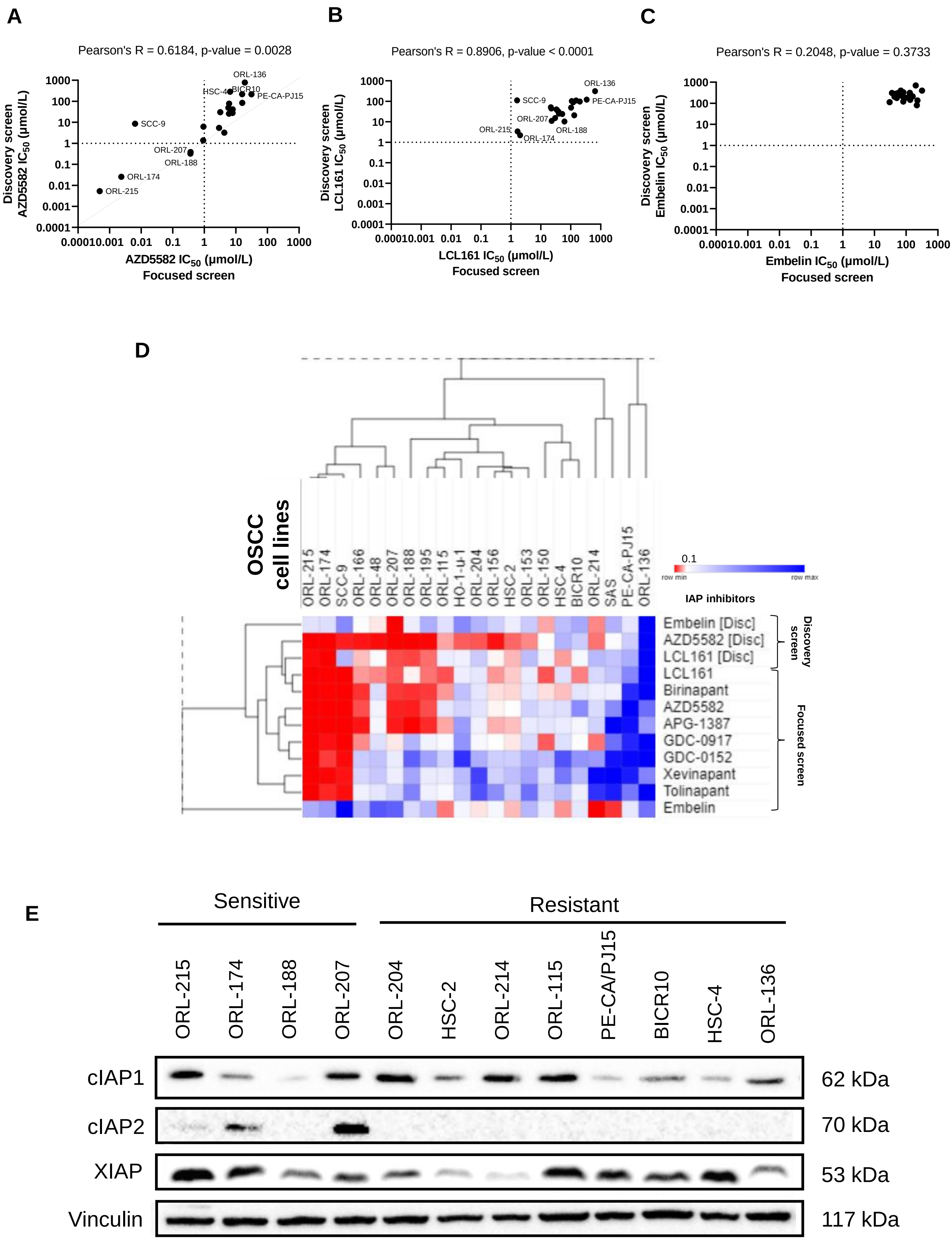

Supplementary Figure 2 – A subset of OSCCs is vulnerable to AZD5582 and other IAP inhibitors.

Correlation plots of IC_{50} for the three overlapping IAP inhibitors in the discovery screen (y-axis) and focus screen (x-axis), for (A) AZD5582; (B) LCL161; and (C) Embelin. (D) Heatmap showing the sensitivity profile of 21 OSCC lines towards the three IAP inhibitors (AZD5582, LCL161 and Embelin) present in the discovery screen, and all nine IAP inhibitors in focus screen. (E) Baseline expression of AZD5582's putative drug target – cIAP1, cIAP2 and XIAP in OSCC lines shown by Western blotting.
