# Supplementary material for: High TNF and NF-κB Pathway Dependency Are Associated with AZD5582 Sensitivity in OSCC via CASP8-Dependent Apoptosis
Source: Cancer Res Commun. 2024 Nov 11;4(11):2919–32. doi: 10.1158/2767-9764.CRC-24-0136 (PMC11551840; doi:10.1158/2767-9764.CRC-24-0136)
Supplement: Supplementary Figure 3 — Correlation of AZD5582 sensitivity with OSCC essential genes. [file crc-24-0136_supplementary_figure_3_suppsf3.pdf]

Supplementary Figure 3

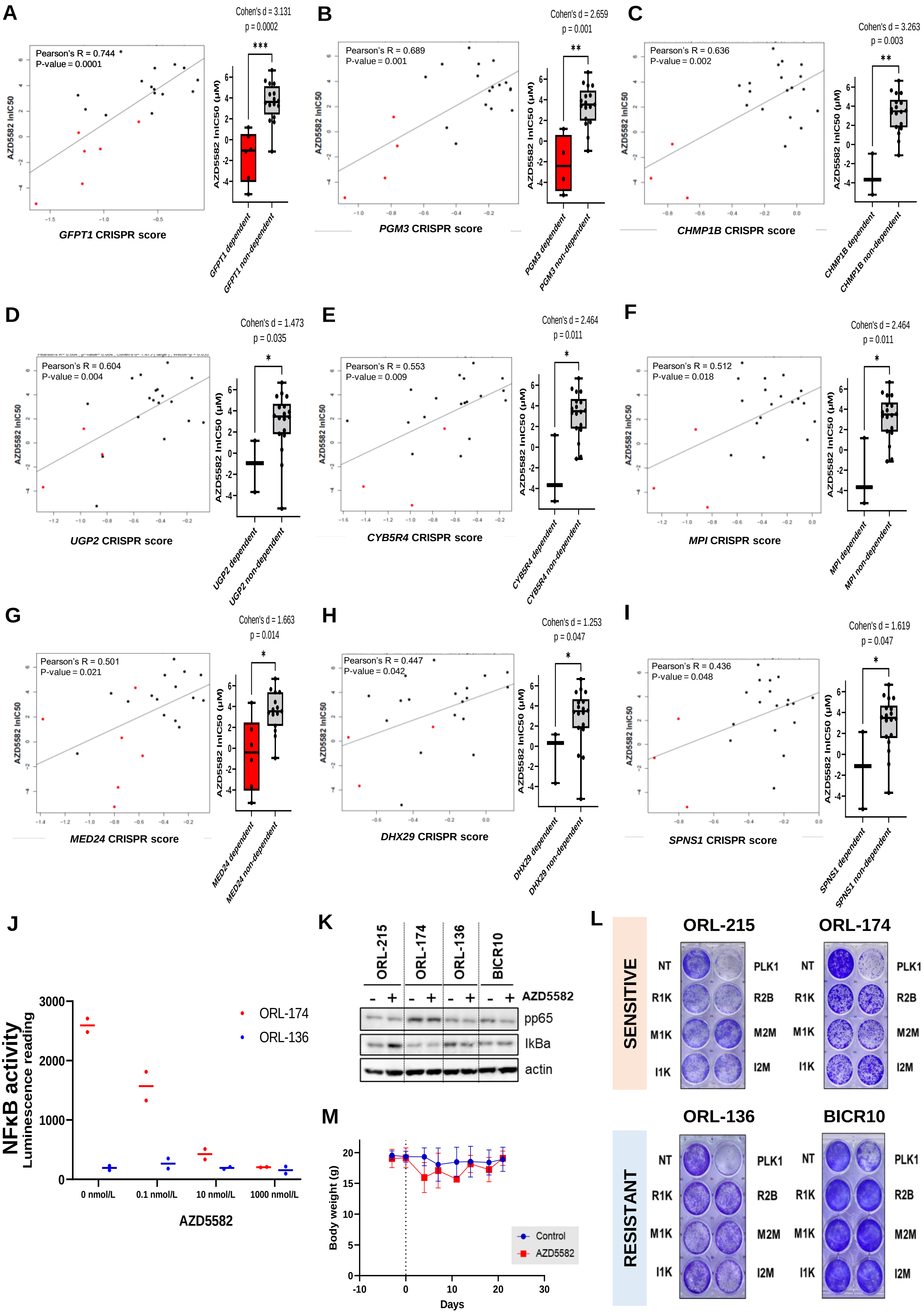

Supplementary Figure 3 – Correlation of AZD5582 sensitivity with OSCC essential genes.

Correlation plots of AZD5582 ln IC_{50} with CRISPR score of essential genes and box plots of dependent versus non-dependent lines for

(A) *GFPT1*; (B) *PGM3*; (C) *CHMP1B*; (D) *UGP2*; (E) *CYB5R4*; (F) *MPI*; (G) *MED24*; (H) *DHX29*; (I) *SPNS1*. Sensitive lines are indicated in red.

(J) NFκB activity as measured by luminescence reading in dependent and non-dependent OSCCs before and after treatment of AZD5582.

(K) Level of p-p65 and IkBa proteins at baseline and upon 15 minutes stimulation with 10 μM AZD5582, as shown by Western blotting.

(L) Colony formation assays of AZD5582-sensitive and –resistant OSCCs upon knockout of *RNF31*, *MAP3K7* and *IKBKG*.

(M) Body weight (g) of mice in control and AZD5582-treated group harboring ORL-207 xenografts.
