# Supplementary material for: High TNF and NF-κB Pathway Dependency Are Associated with AZD5582 Sensitivity in OSCC via CASP8-Dependent Apoptosis
Source: Cancer Res Commun. 2024 Nov 11;4(11):2919–32. doi: 10.1158/2767-9764.CRC-24-0136 (PMC11551840; doi:10.1158/2767-9764.CRC-24-0136)
Supplement: Supplementary Figure 4 — Investigation of gene expression changes associated with AZD5582. [file crc-24-0136_supplementary_figure_4_suppsf4.pdf]

Supplementary Figure 4

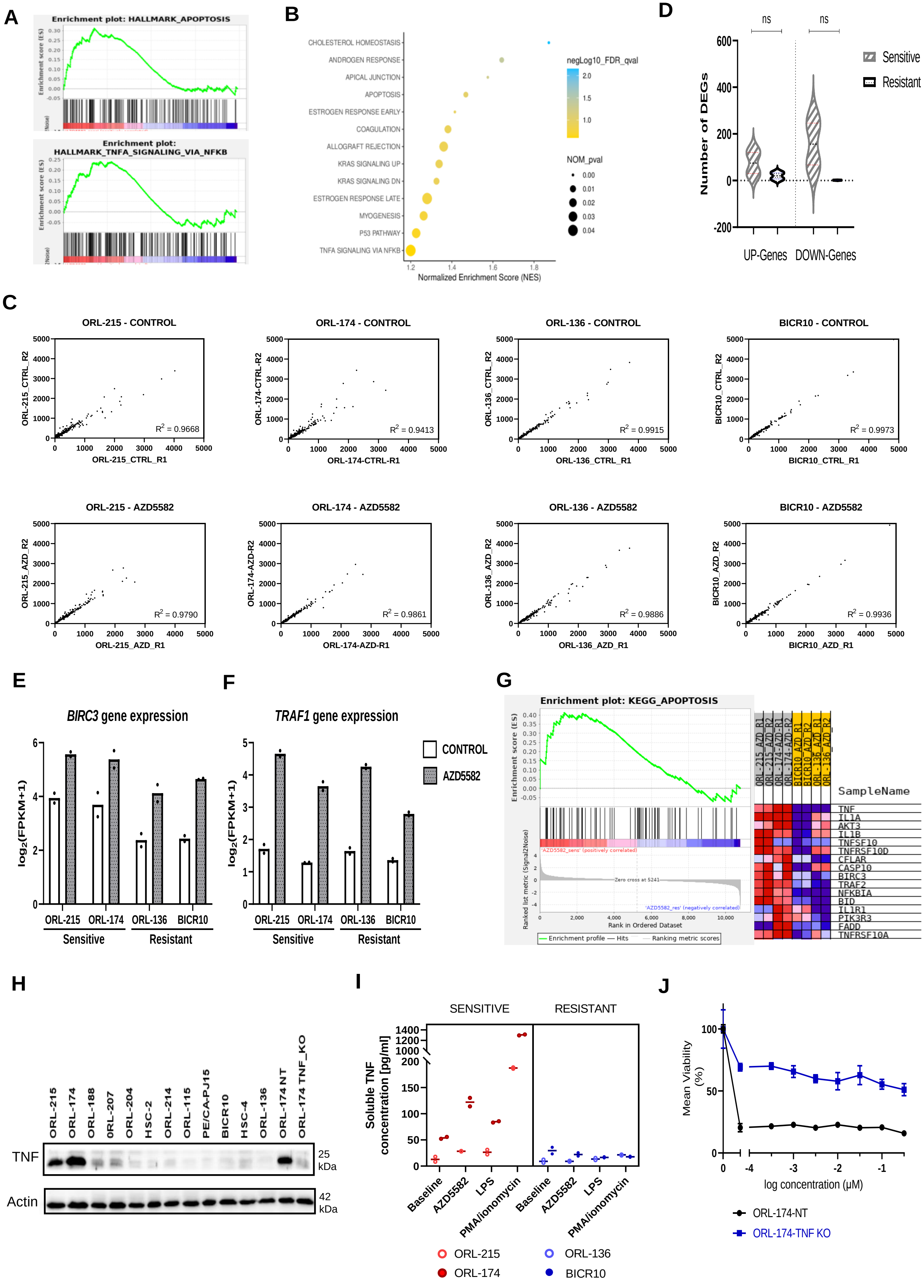

Supplementary Figure 4 – Investigation of gene expression changes associated with AZD5582.

(A) Gene set enrichment analysis (GSEA) plot of two hallmarks – apoptosis and TNFα signaling via NF-κB pathway that are enriched among AZD5582-sensitive OSCC cell lines (ORL-215, ORL-174), as compared with AZD5582-resistant OSCC (ORL-136, BICR10).

(B) Gene set enrichment analysis showing hallmarks enriched among AZD5582-sensitive OSCC lines, with normalized p-value < 0.05.

(C) Violin plots of differentially expressed genes (DEGs) numbers among AZD5582-sensitive and –resistant OSCC.

(D) Correlation plots of the two biological replicates (x-axis: R1 & y-axis: R2) of the RNA-seq data using $\log_2$(FPKM+1). Top rows were control, bottom rows were AZD5582-treated.

(E) Plot showing the upregulation of *BIRC3* gene expression upon AZD5582 treatment.

(F) Plot showing the upregulation of *TRAF1* gene expression upon AZD5582 treatment.

(G) GSEA plot comparing AZD5582-treated sensitive and resistant lines, with KEGG pathway of apoptosis being among the most enriched. Heatmap shows the pathway members with higher gene expression among AZD5582-sensitive OSCC.

(H) Western blot showing the level of TNF being more highly expressed among AZD5582-sensitive OSCC lines.

(I) Soluble TNF secreted in the conditioned media of AZD5582-sensitive and –resistant OSCC lines were measured using enzyme-linked immunosorbent assay (ELISA)

(J) Dose response curve of AZD5582 in ORL-174 with or without TNF knockout (TNF KO).
