# Supplementary material for: High TNF and NF-κB Pathway Dependency Are Associated with AZD5582 Sensitivity in OSCC via CASP8-Dependent Apoptosis
Source: Cancer Res Commun. 2024 Nov 11;4(11):2919–32. doi: 10.1158/2767-9764.CRC-24-0136 (PMC11551840; doi:10.1158/2767-9764.CRC-24-0136)
Supplement: Supplementary Figure 5 — Necroptosis is activated in the absent of CASP8 upon AZD5582 treatment as shown by induction of p-MLKL. [file crc-24-0136_supplementary_figure_5_suppsf5.pdf]

Supplementary Figure 5

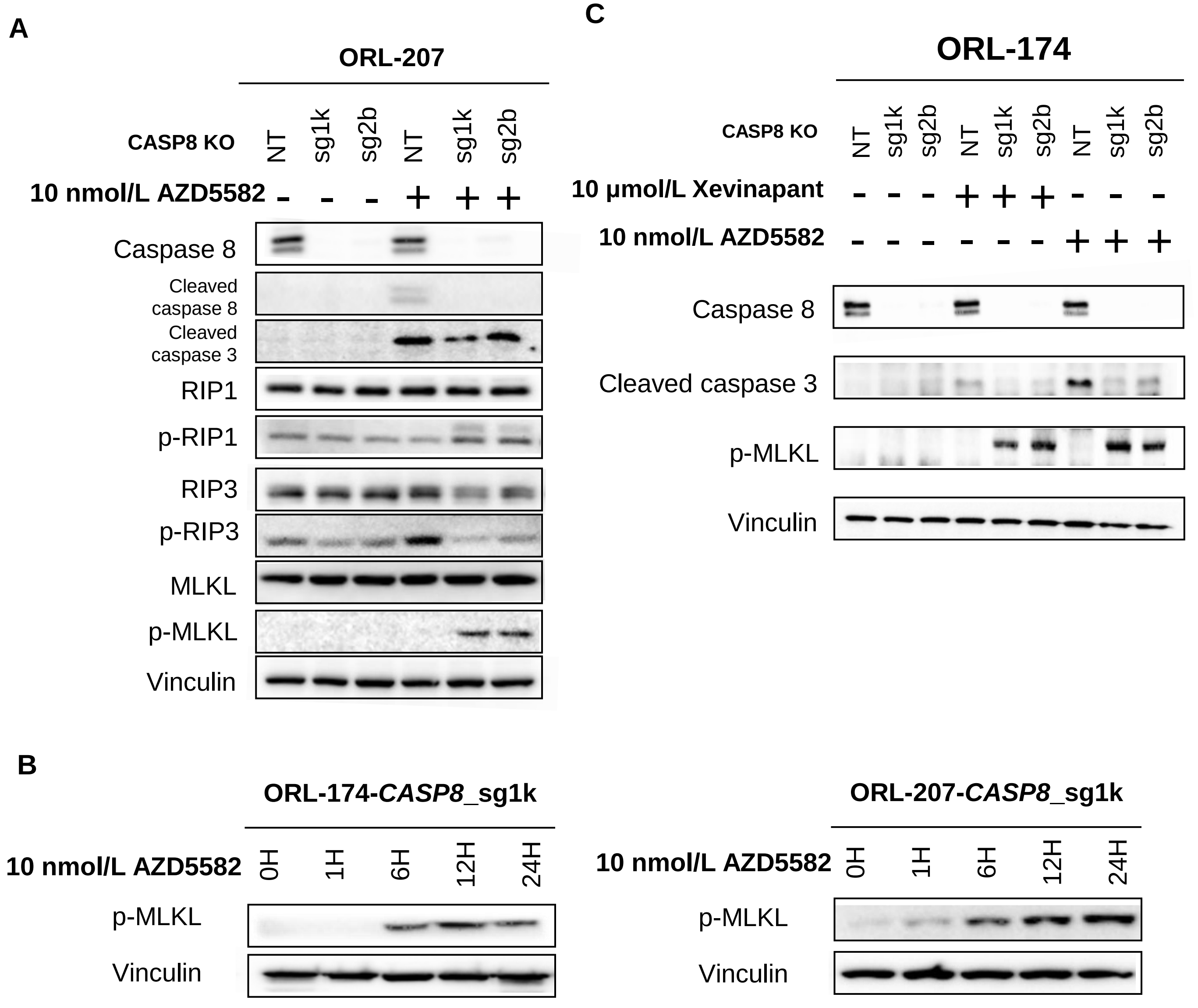

Supplementary Figure 5 – Necroptosis is activated in the absent of CASP8 upon AZD5582 treatment as shown by induction of p-MLKL.

(A) Western blot showing the level of apoptosis and necroptosis marker in ORL-207 with or without CASP8 knockout.

(B) Gradual induction of p-MLKL level observed in the CASP8-knocked out ORL-174 and ORL-207.

(C) Western blot showing that the switch to necroptosis in CASP8-knocked out ORL-174 is not unique to AZD5582, but also seen when another IAP inhibitor, xevinapant was used.
