# Supplementary material for: High TNF and NF-κB Pathway Dependency Are Associated with AZD5582 Sensitivity in OSCC via CASP8-Dependent Apoptosis
Source: Cancer Res Commun. 2024 Nov 11;4(11):2919–32. doi: 10.1158/2767-9764.CRC-24-0136 (PMC11551840; doi:10.1158/2767-9764.CRC-24-0136)

Supplementary Figure 6 – All uncropped western blot images

For Fig. 2F, S.Fig. 2E

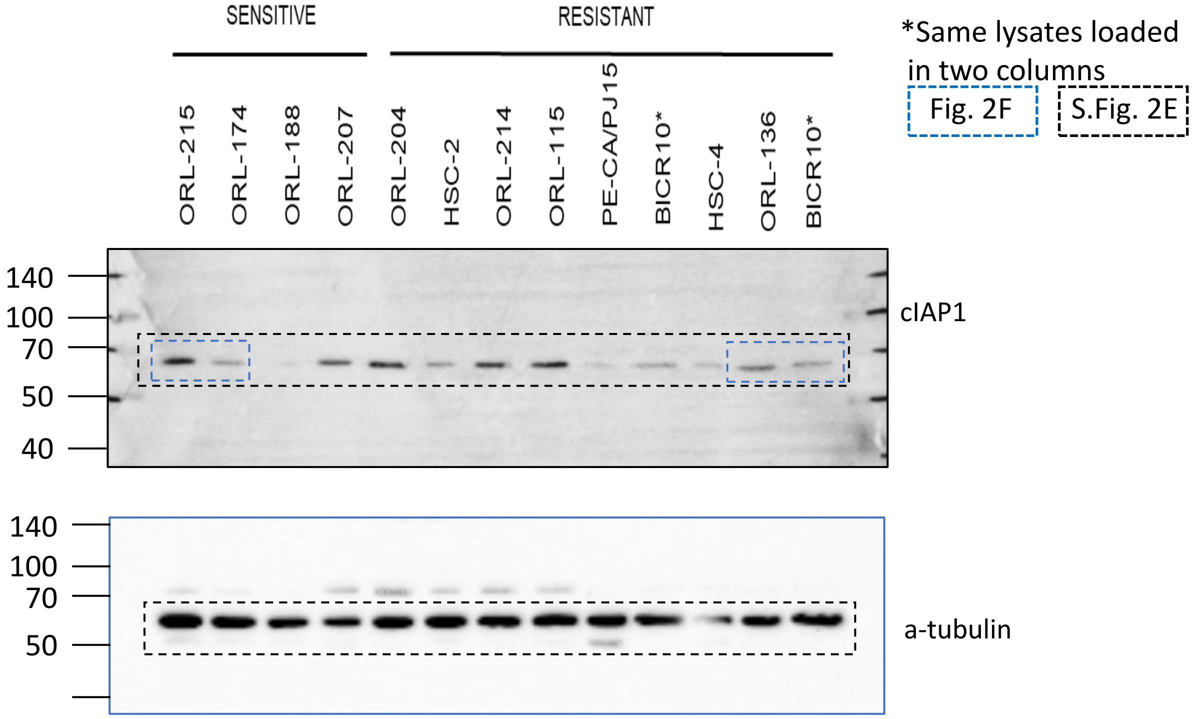

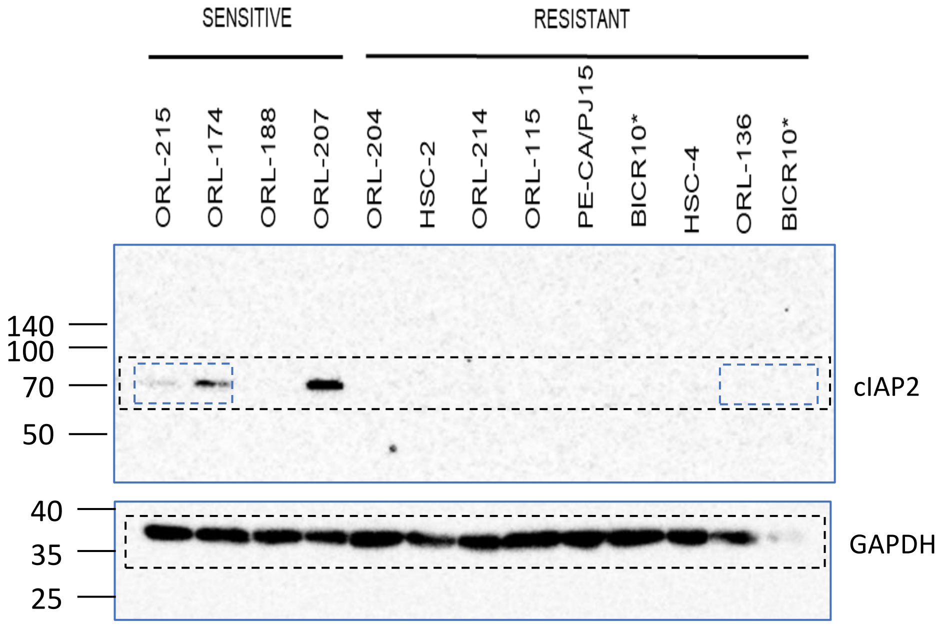

Supplementary Figure 6 – All uncropped western blot images

For Fig. 2F, S.Fig. 2E

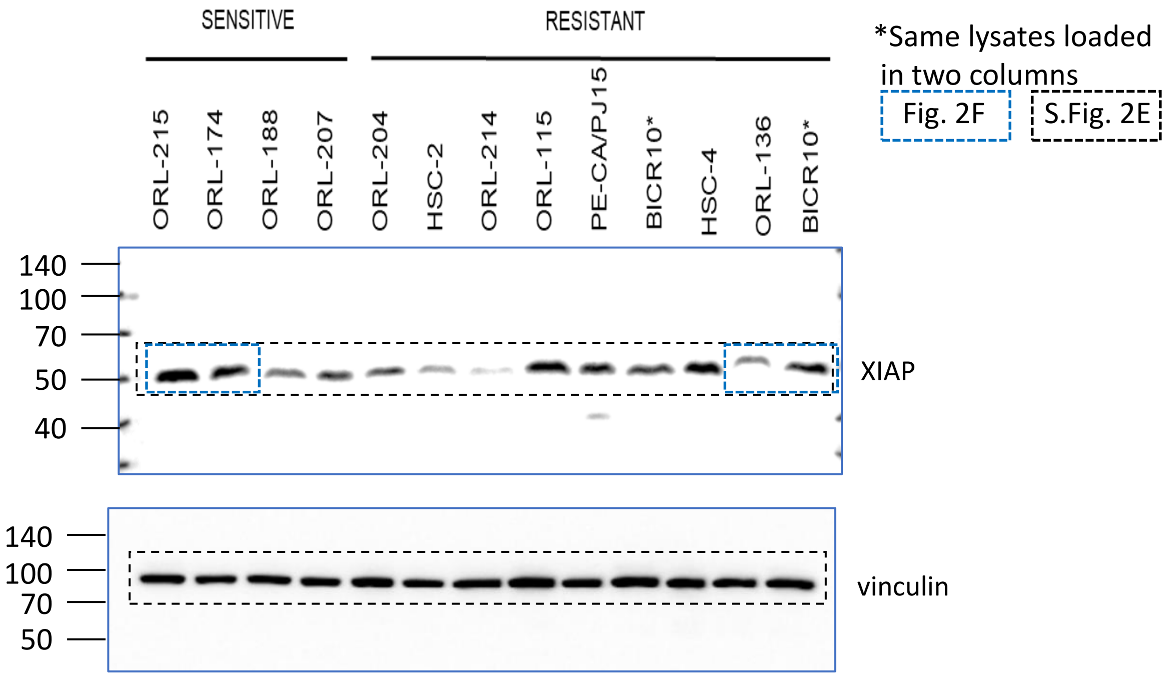

Supplementary Figure 6 – All uncropped western blot images

For Fig. 2G

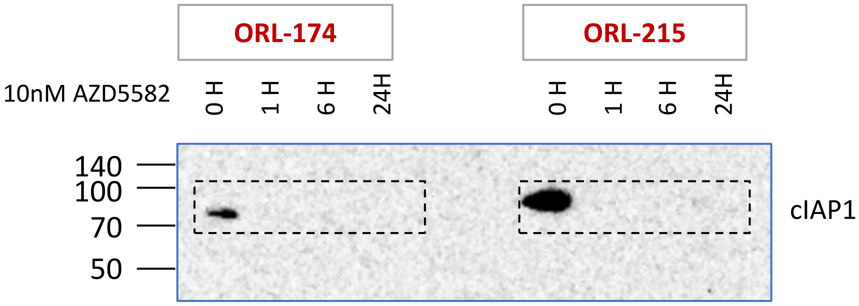

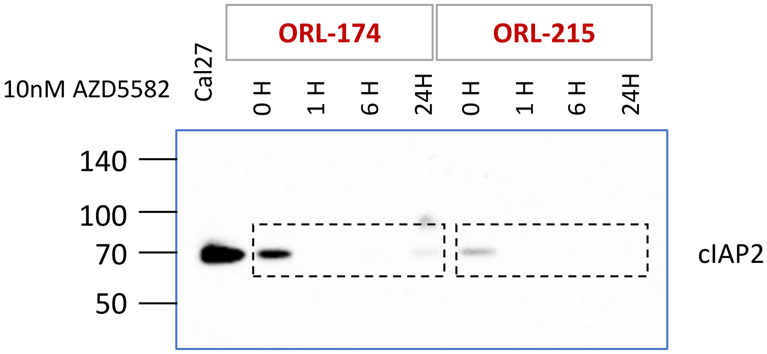

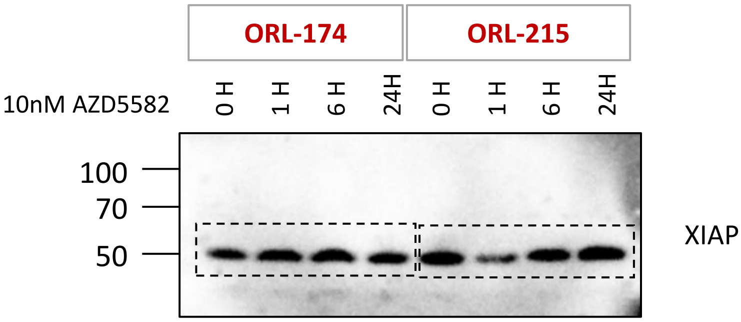

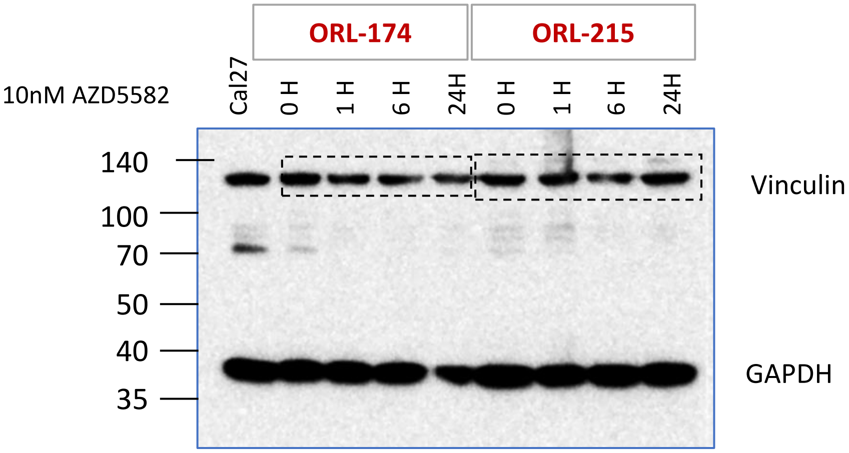

Supplementary Figure 6 – All uncropped western blot images

For Fig. 2G

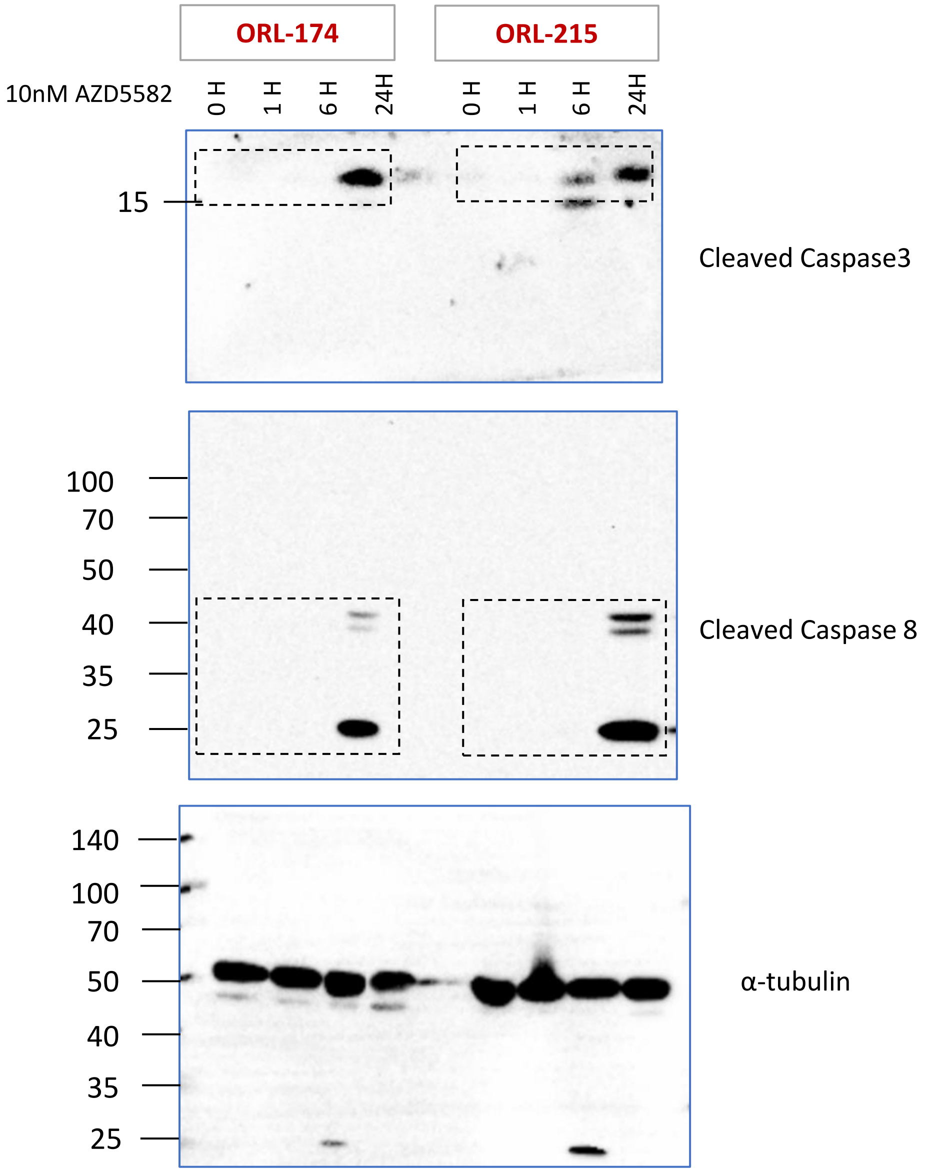

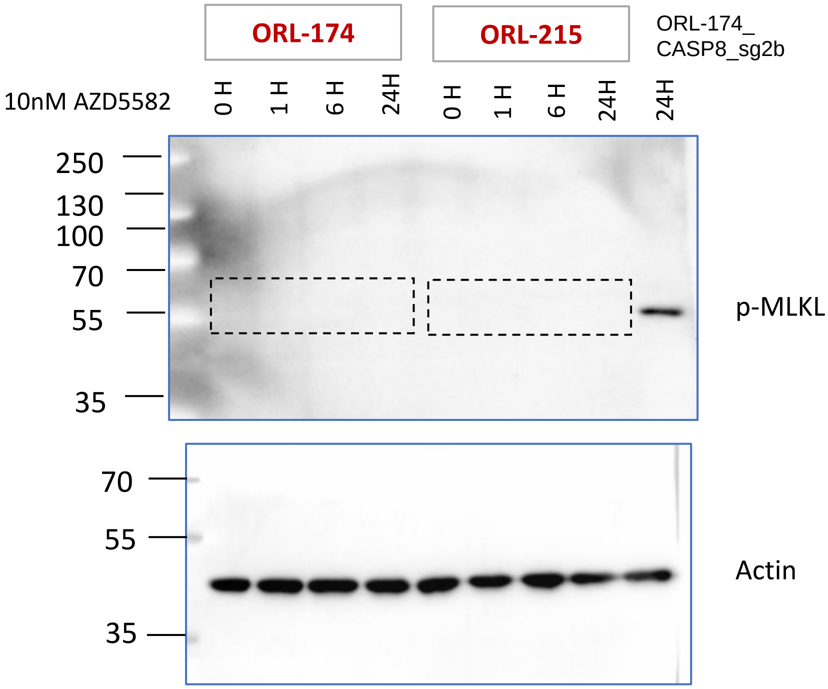

Supplementary Figure 6 – All uncropped western blot images

For Fig. 2G

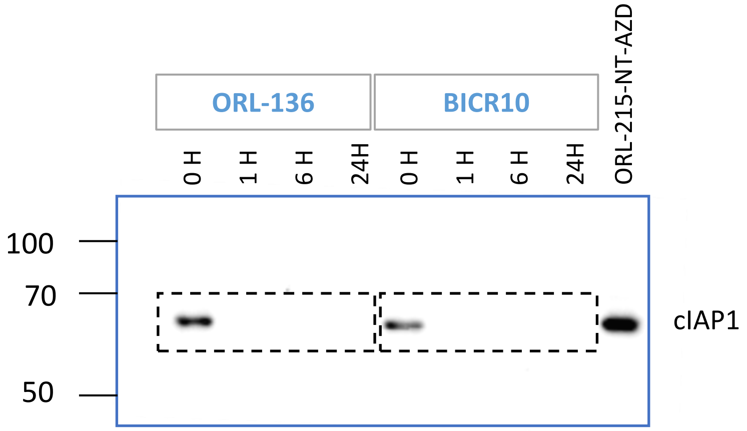

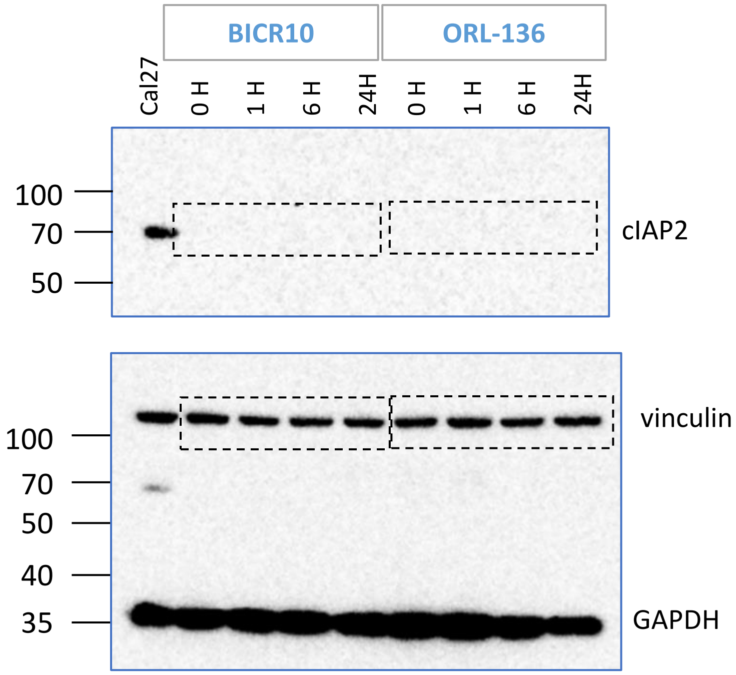

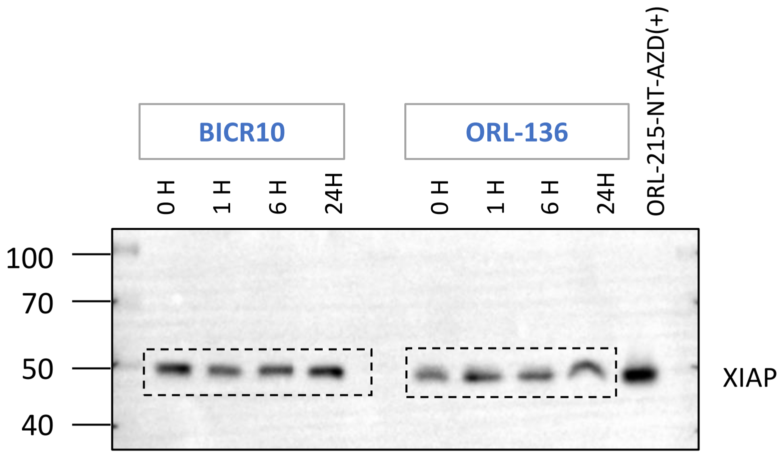

Supplementary Figure 6 – All uncropped western blot images

For Fig. 2G

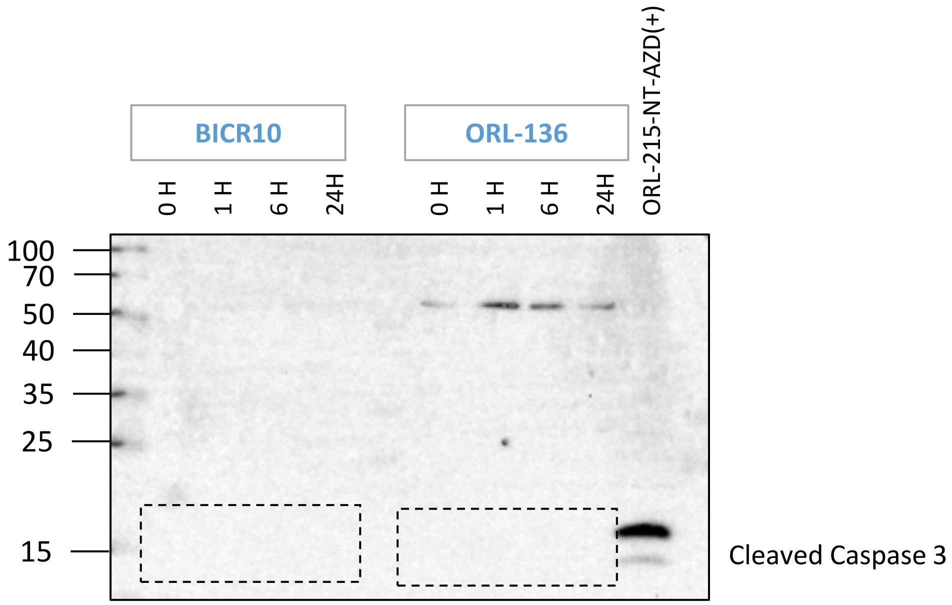

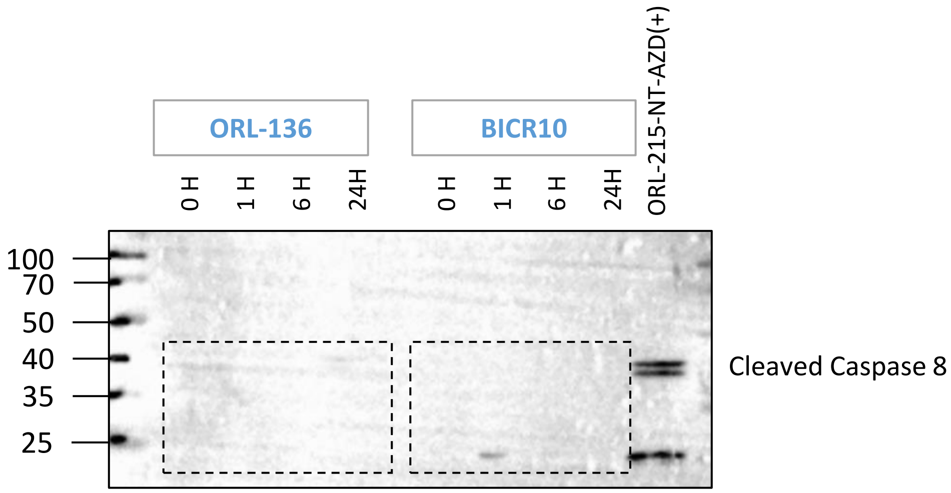

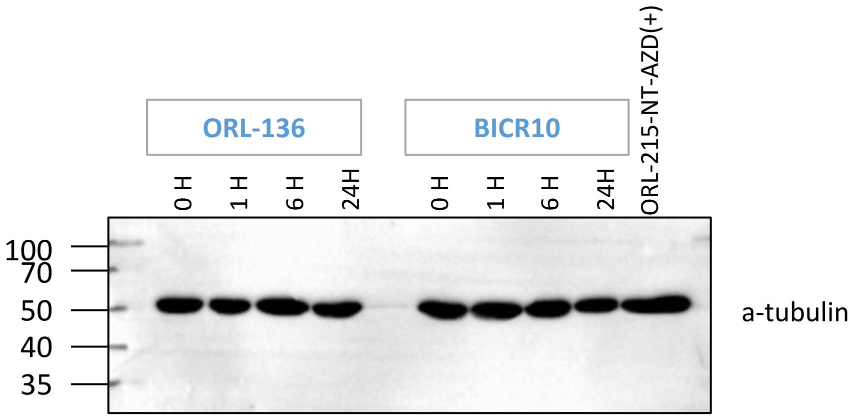

Supplementary Figure 6 – All uncropped western blot images

For Fig. 2G

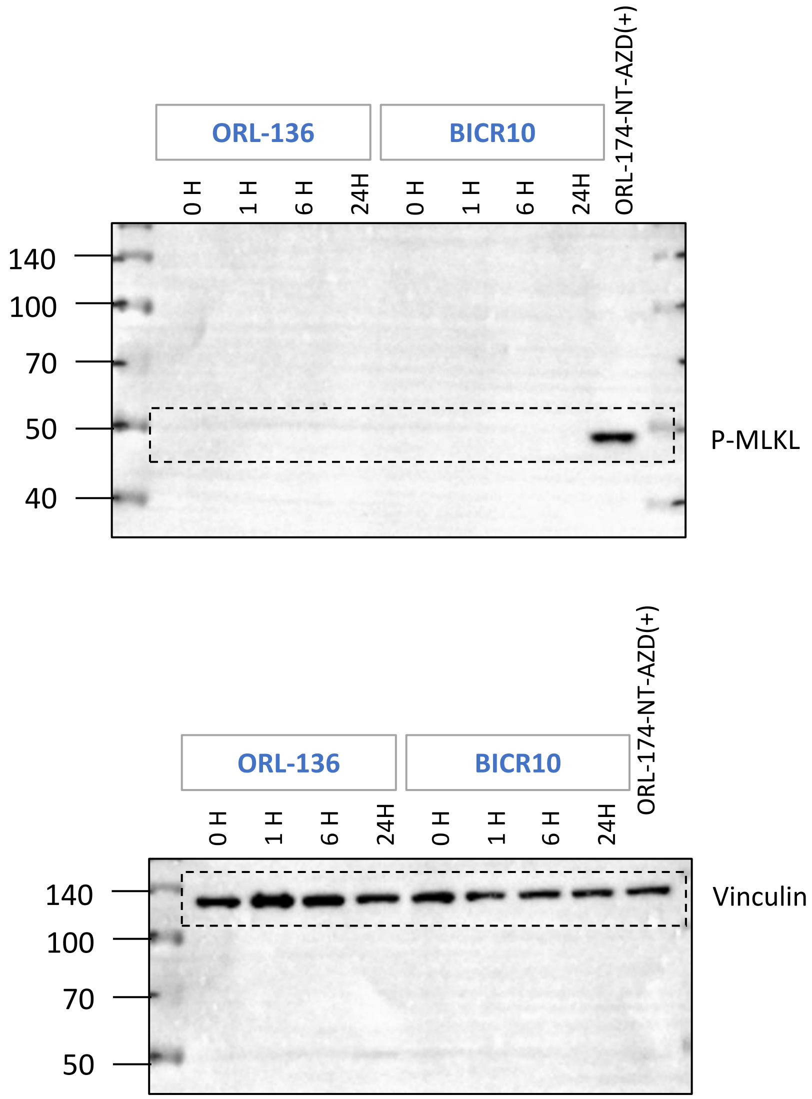

Supplementary Figure 6 – All uncropped western blot images

For Fig. 3D

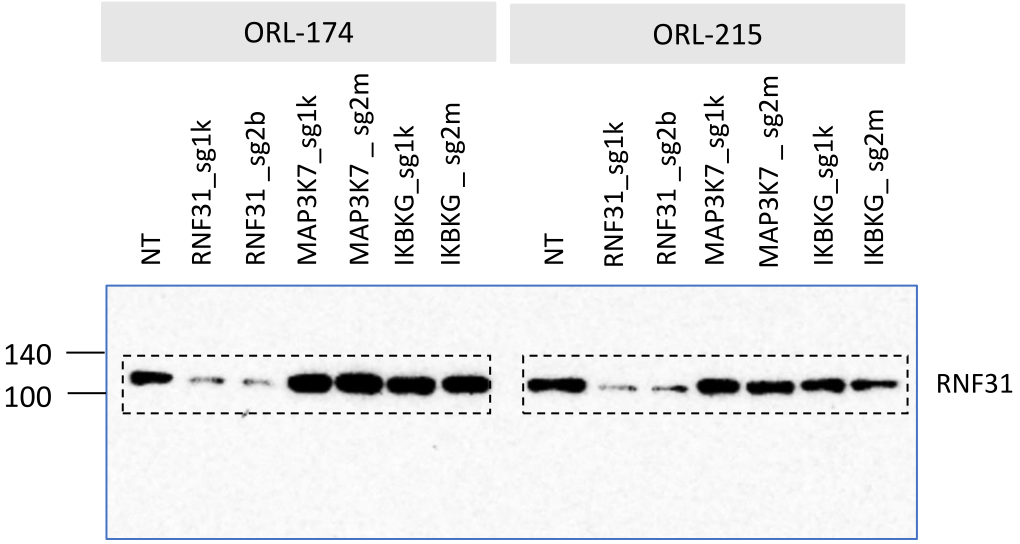

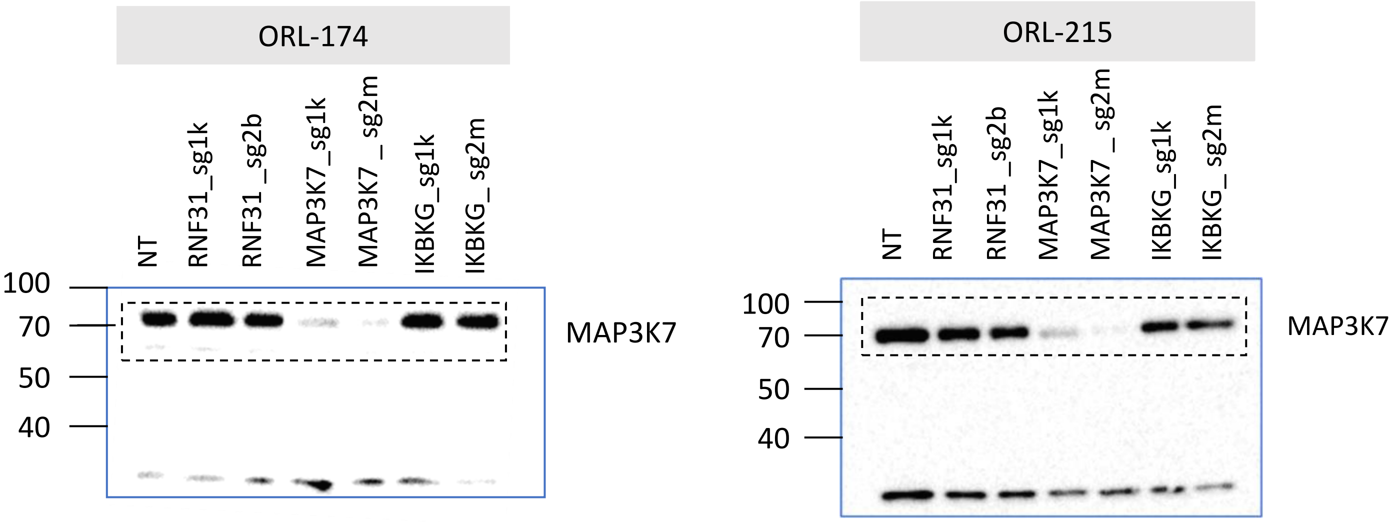

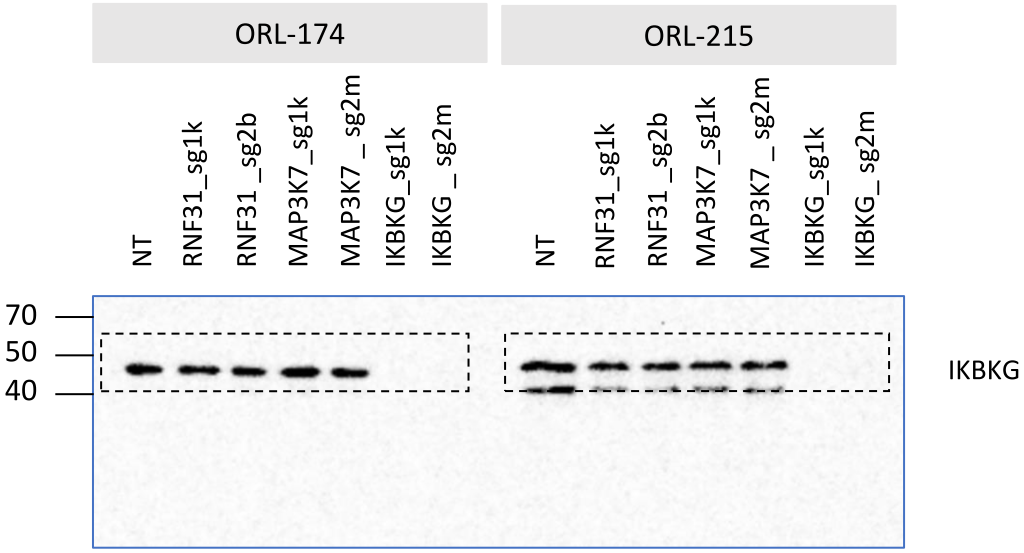

Supplementary Figure 6 – All uncropped western blot images

For Fig. 3D

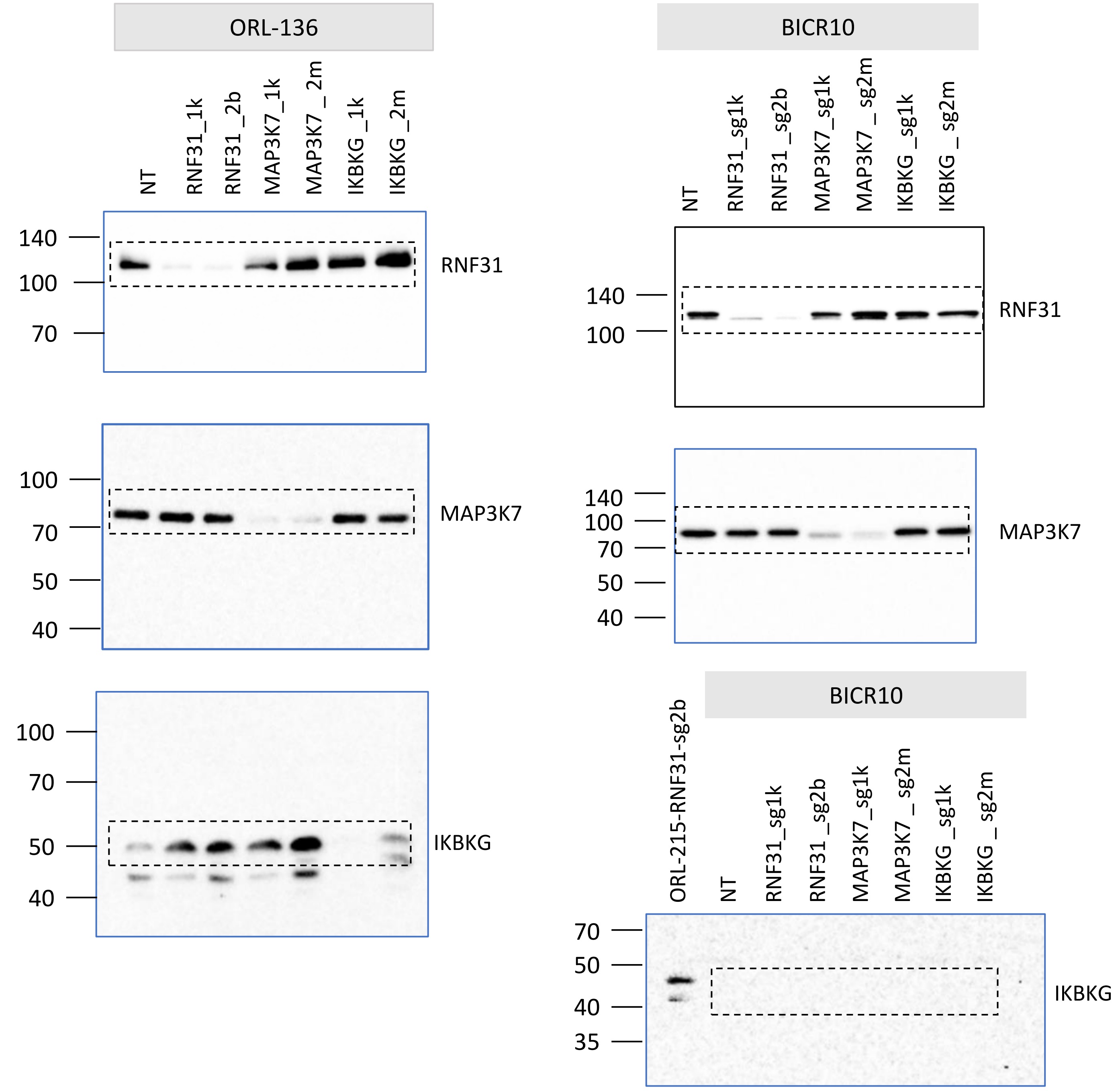

Supplementary Figure 6 – All uncropped western blot images

For Fig. 3D

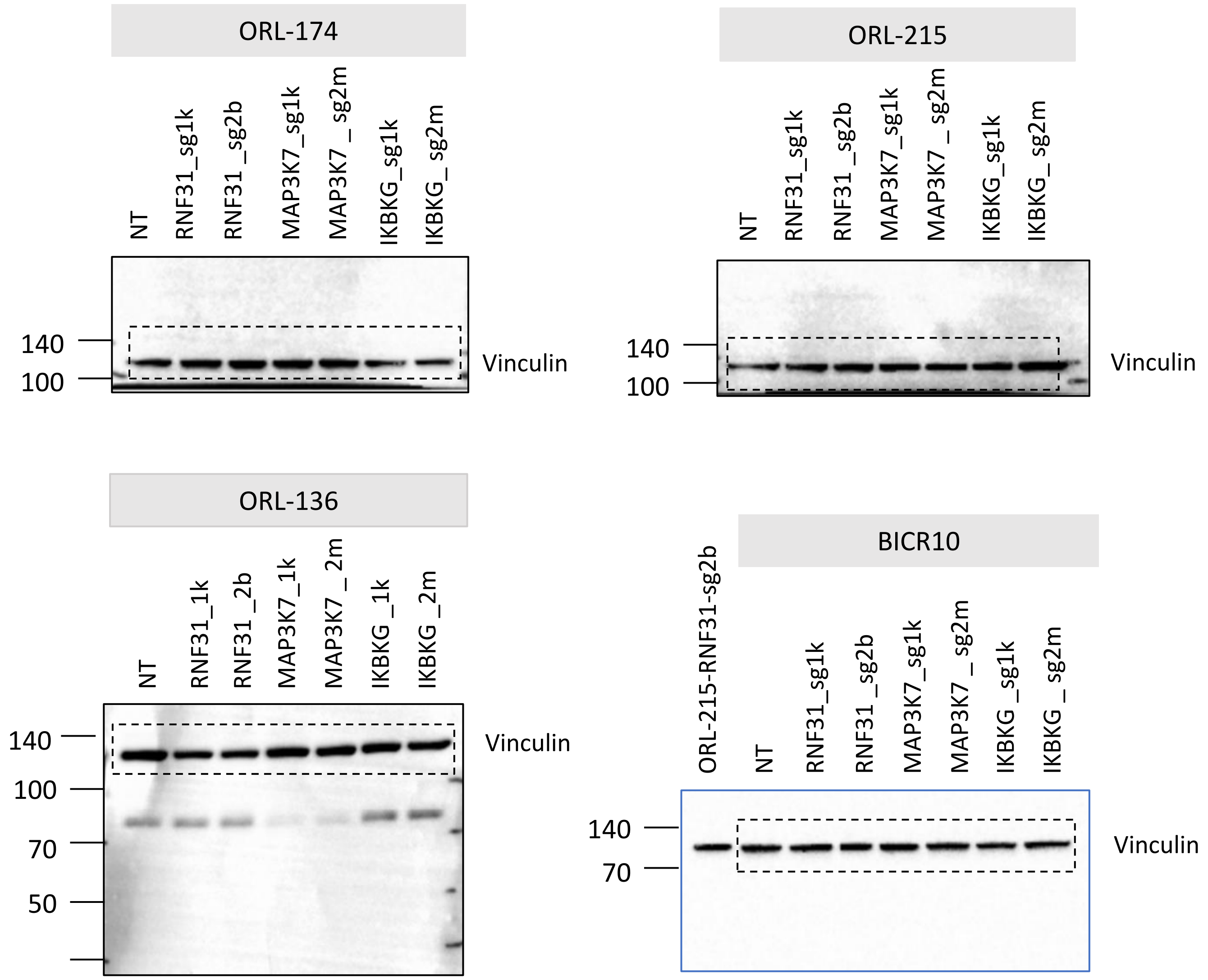

Supplementary Figure 6 – All uncropped western blot images

For Fig. 3H

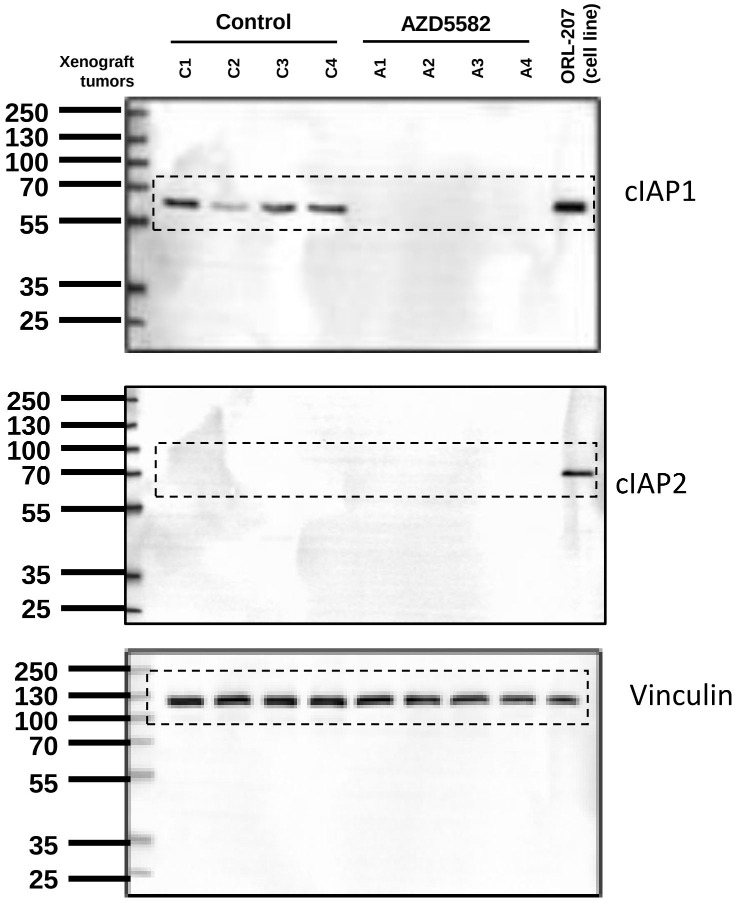

Supplementary Figure 6 – All uncropped western blot images

For Fig. S3K

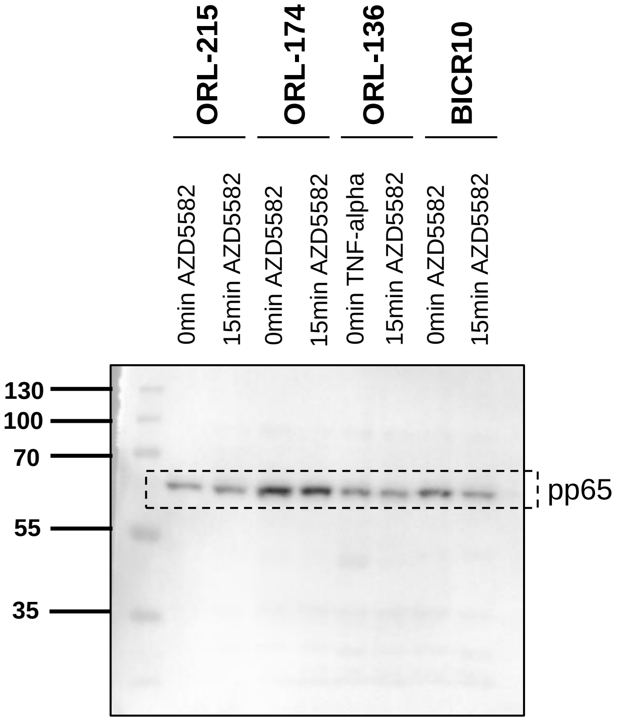

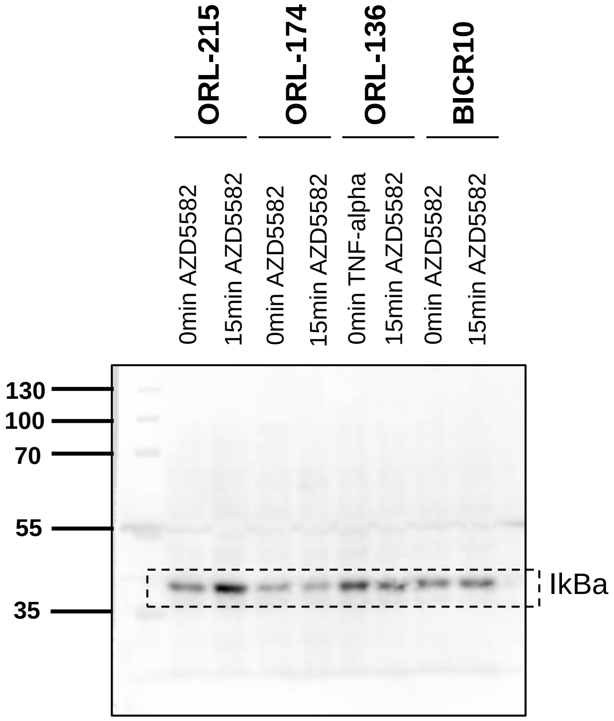

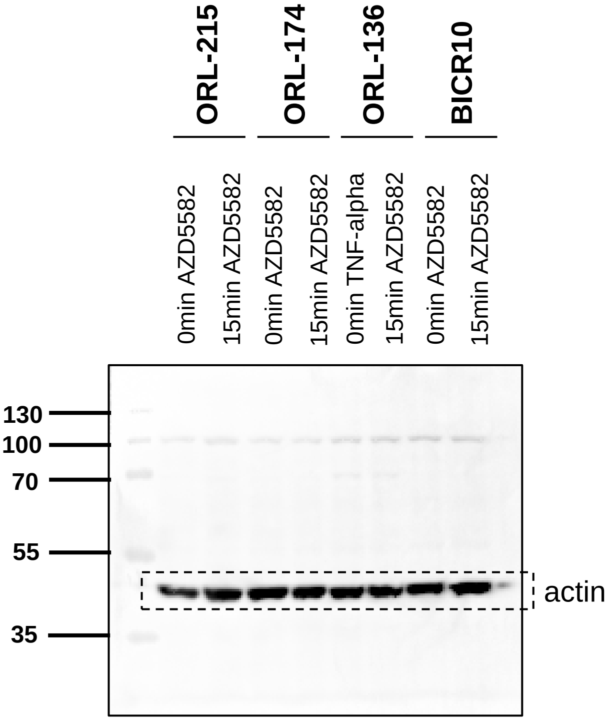

Supplementary Figure 6 – All uncropped western blot images

For Fig. 4G

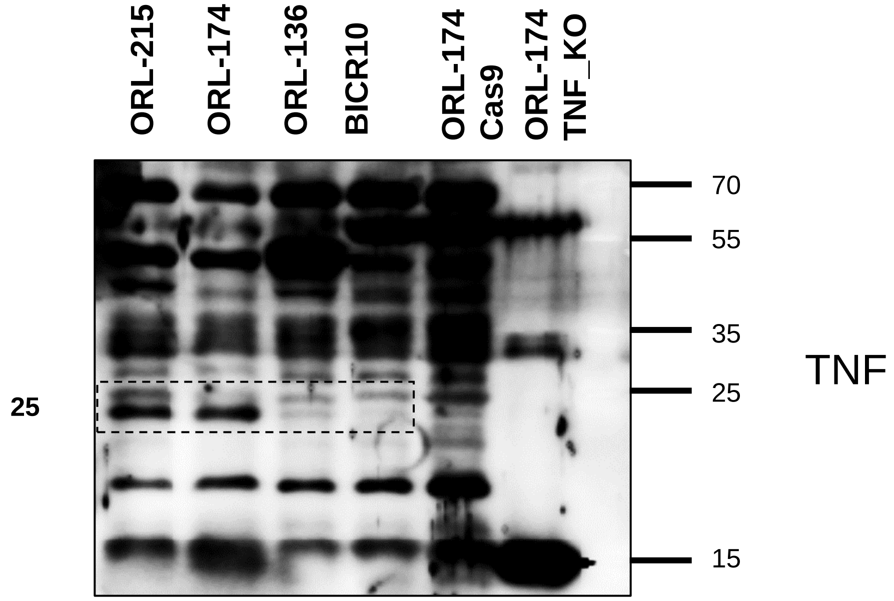

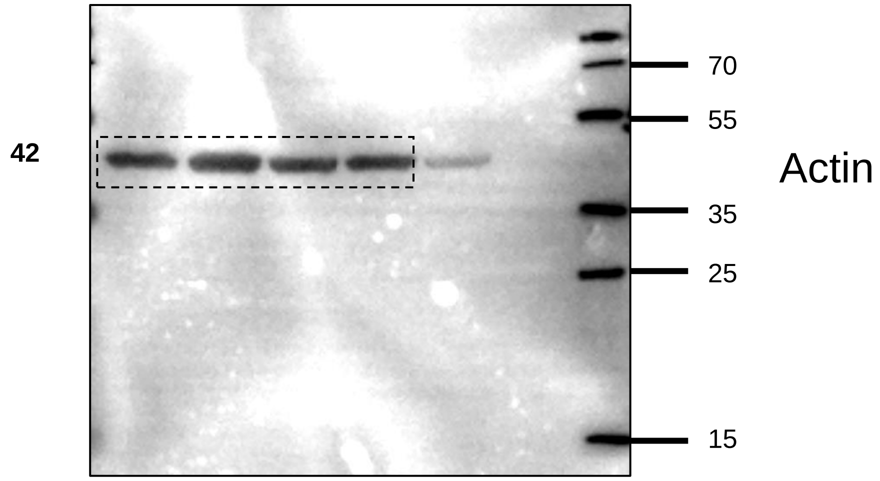

Supplementary Figure 6 – All uncropped western blot images

For Supp. Fig. 4H

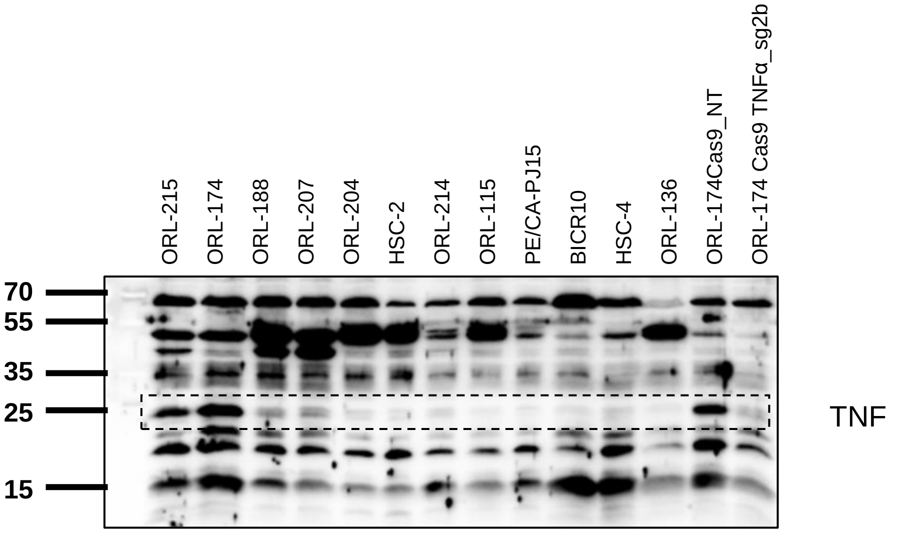

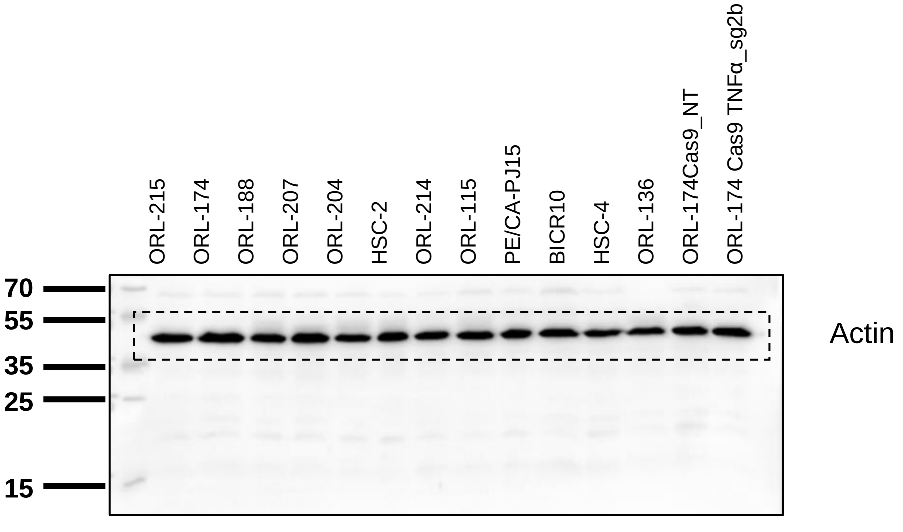

Supplementary Figure 6 – All uncropped western blot images

For Fig. 5A

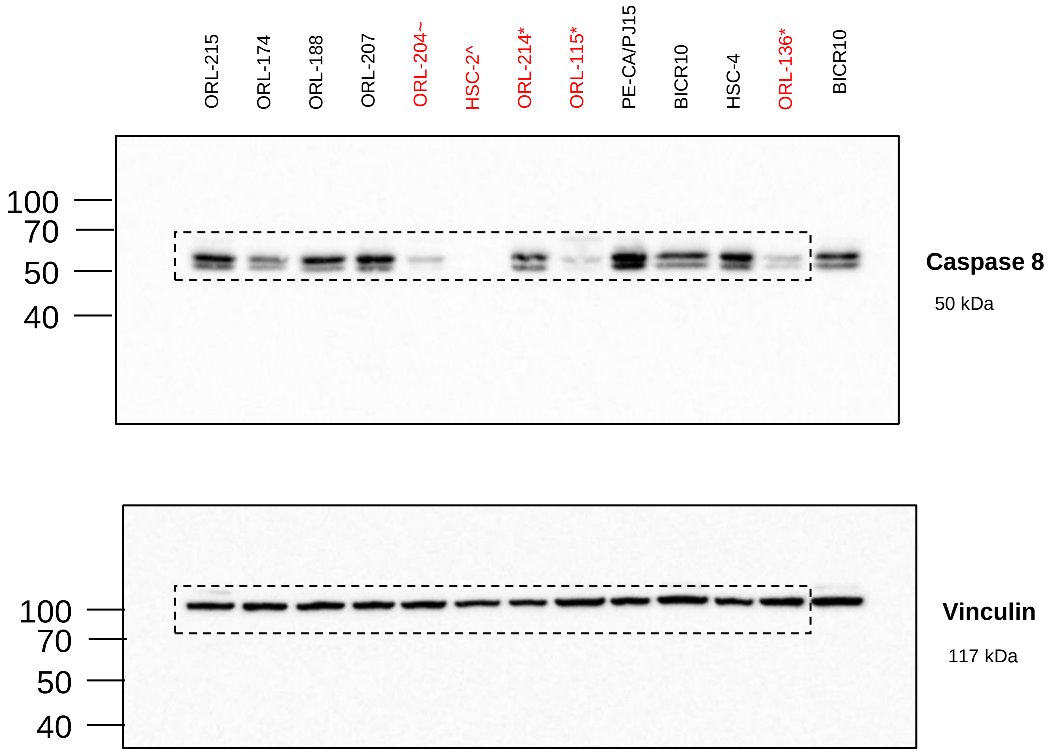

Supplementary Figure 6 – All uncropped western blot images

For Fig. 5D

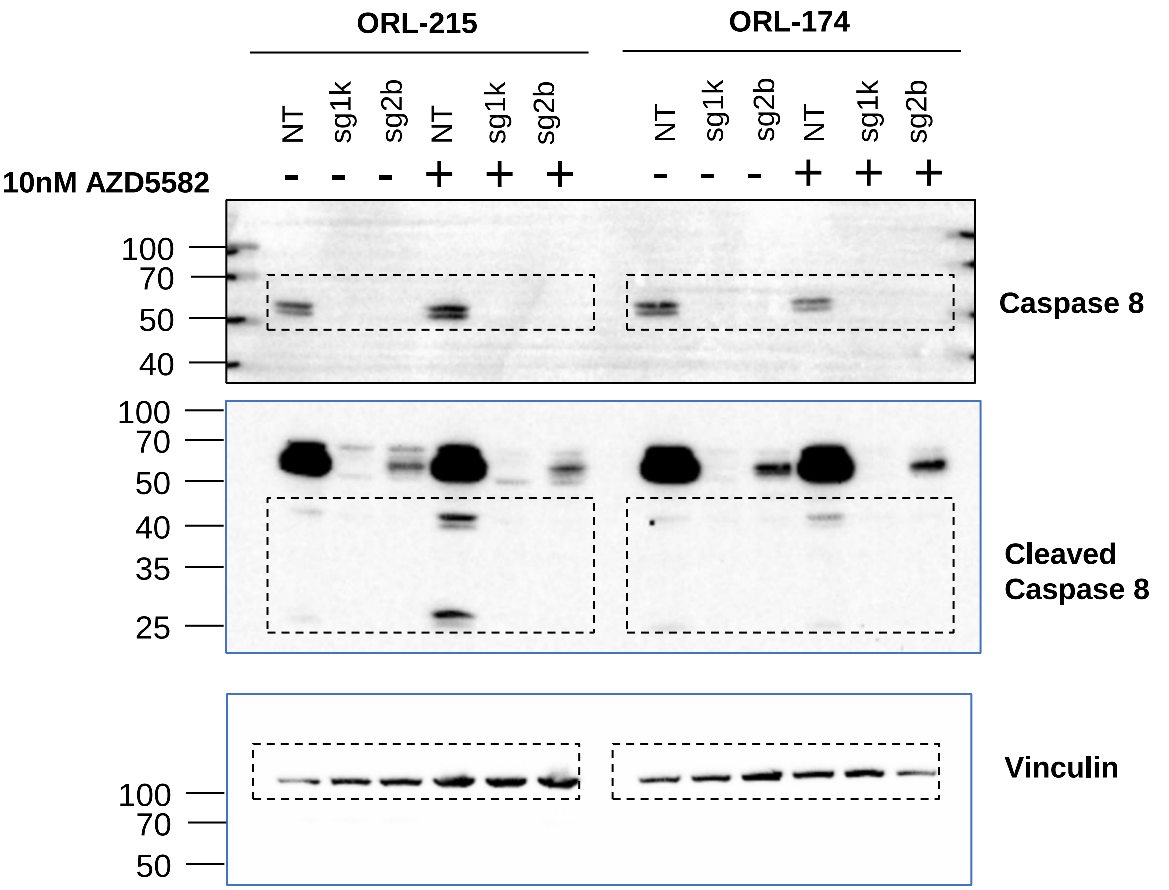

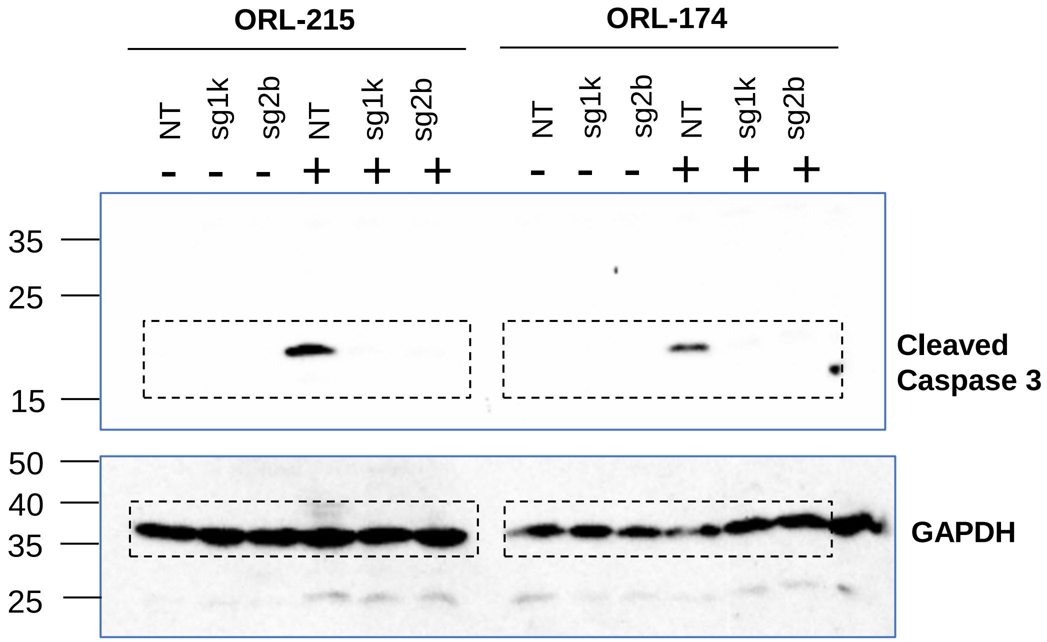

Supplementary Figure 6 – All uncropped western blot images

For Fig. 5D

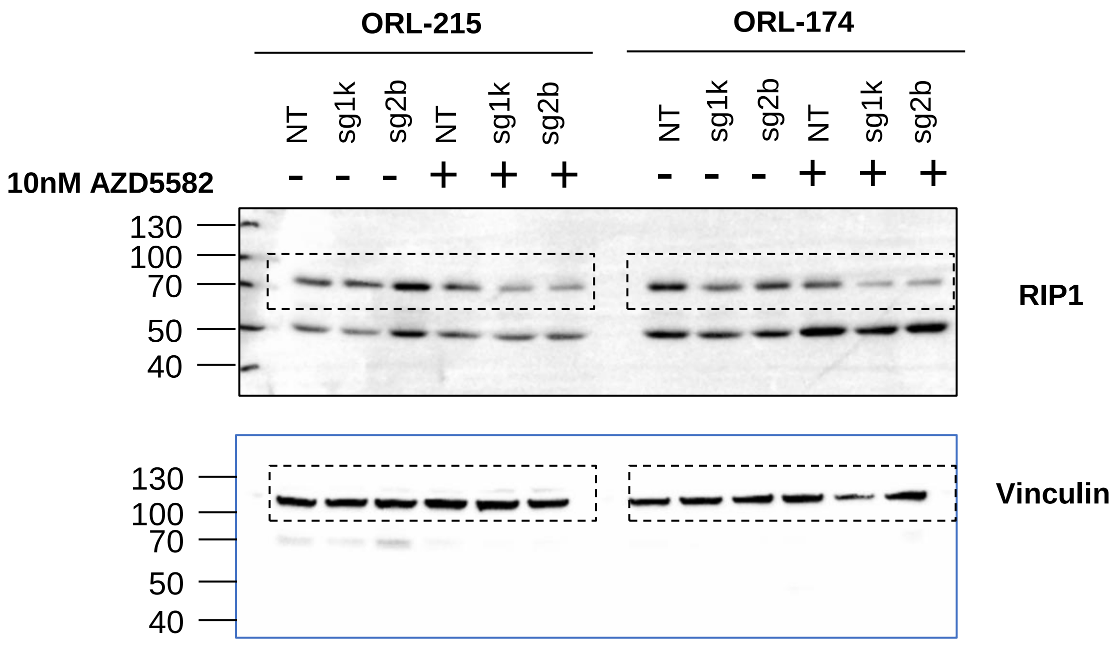

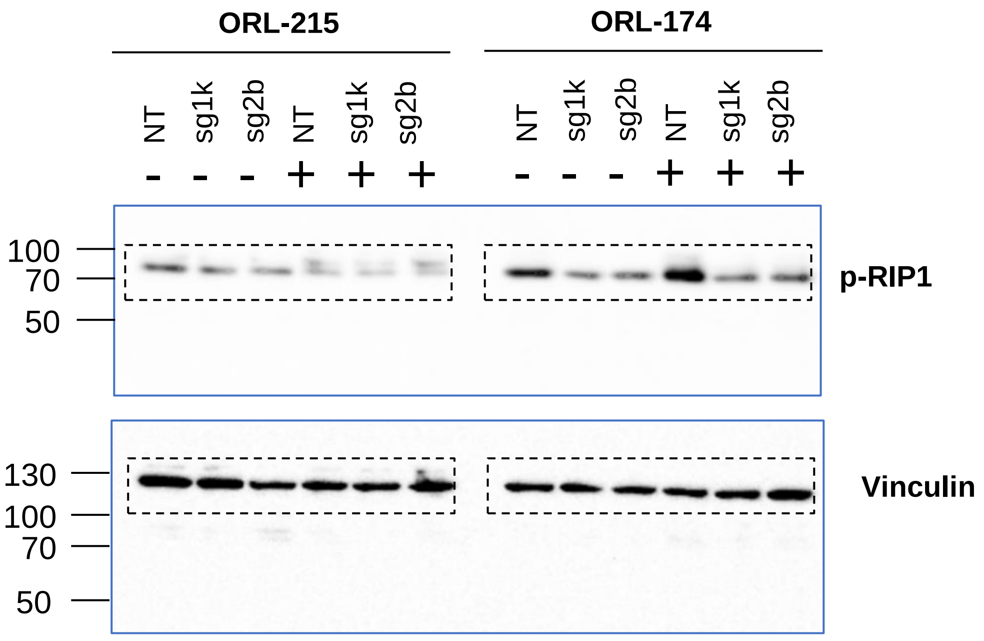

Supplementary Figure 6 – All uncropped western blot images

For Fig. 5D

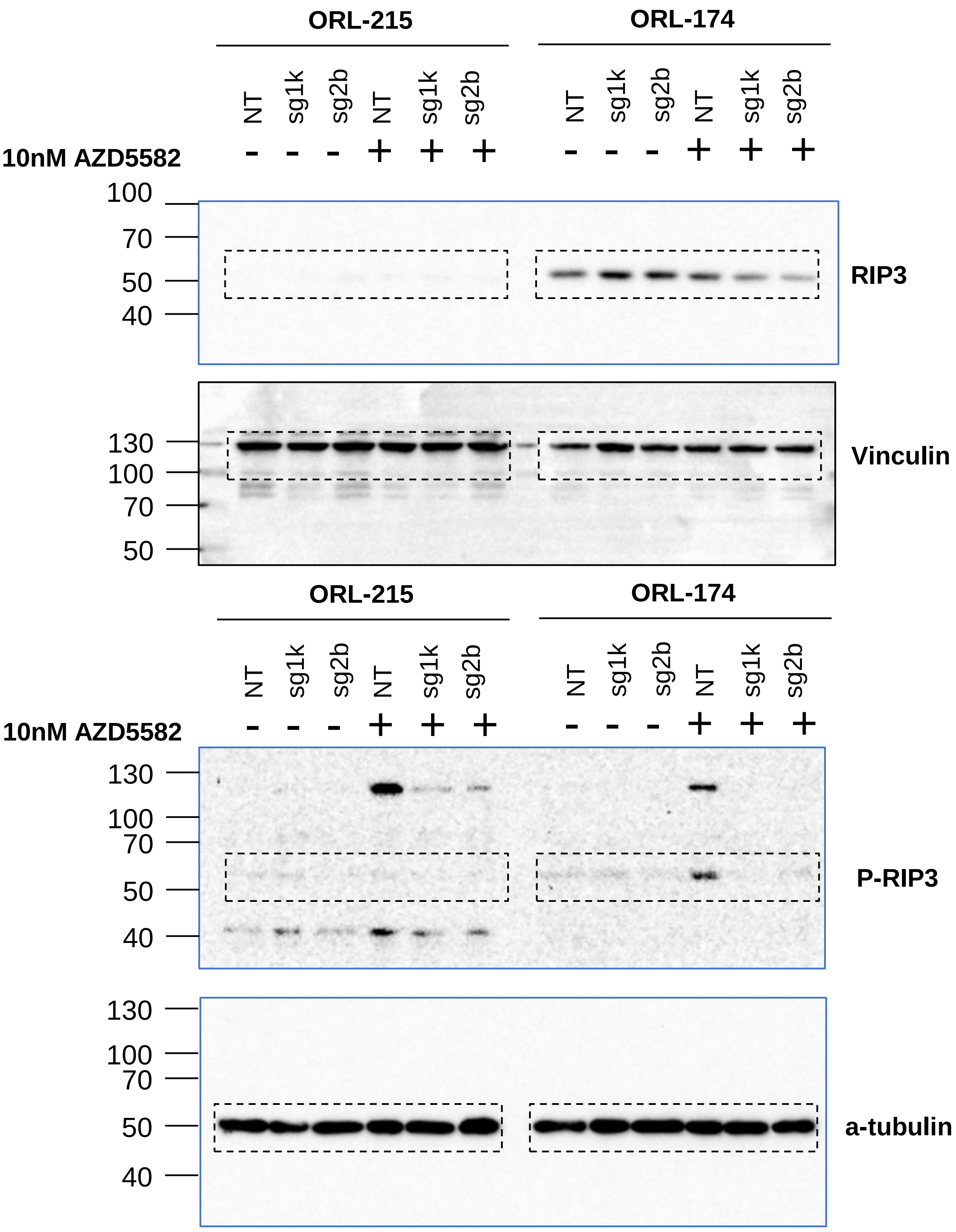

Supplementary Figure 6 – All uncropped western blot images

For Fig. 5D

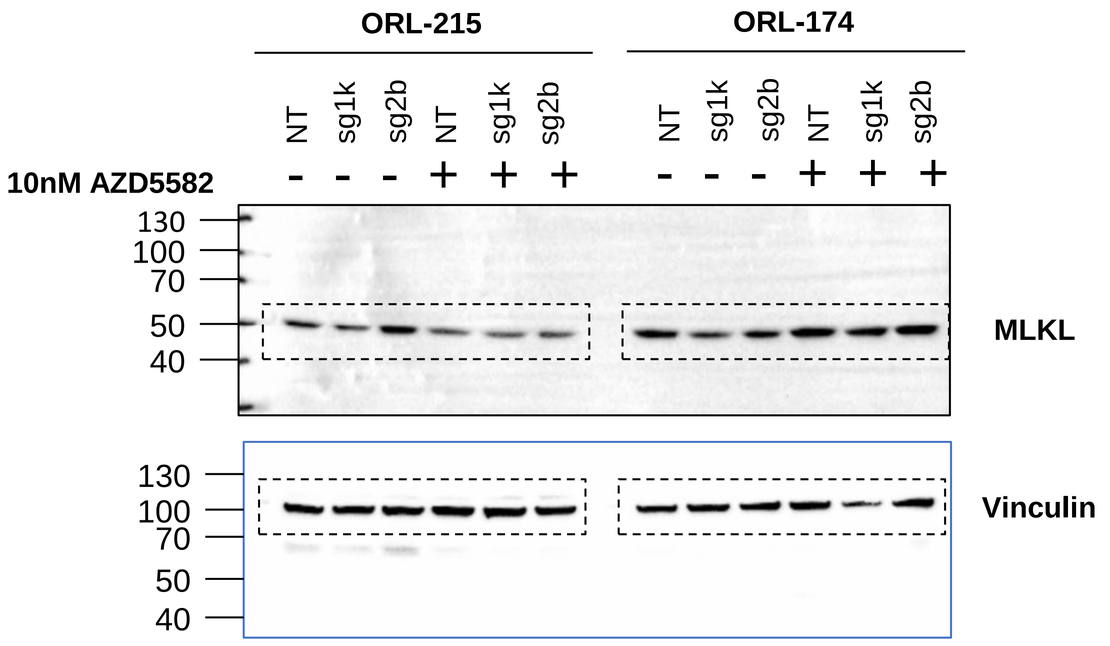

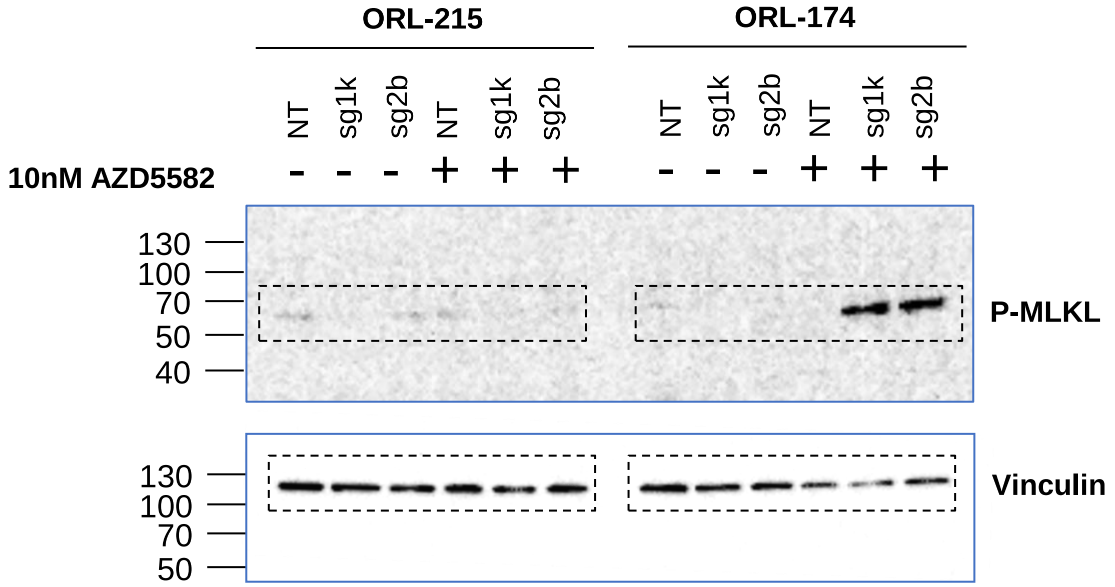

Supplementary Figure 6 – All uncropped western blot images

For Fig. 5F

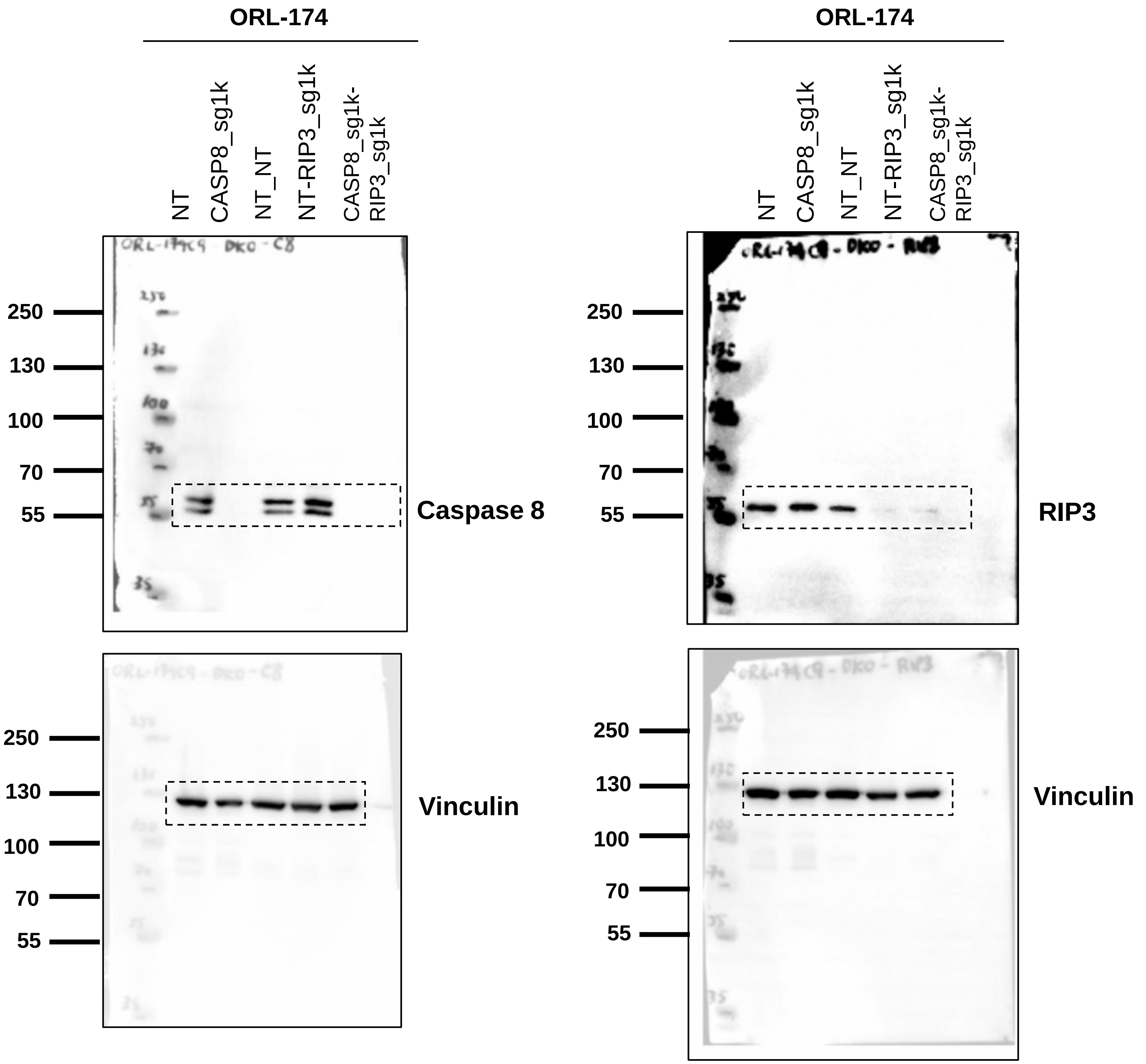

Supplementary Figure 6 – All uncropped western blot images

For Supp. Fig. 5A

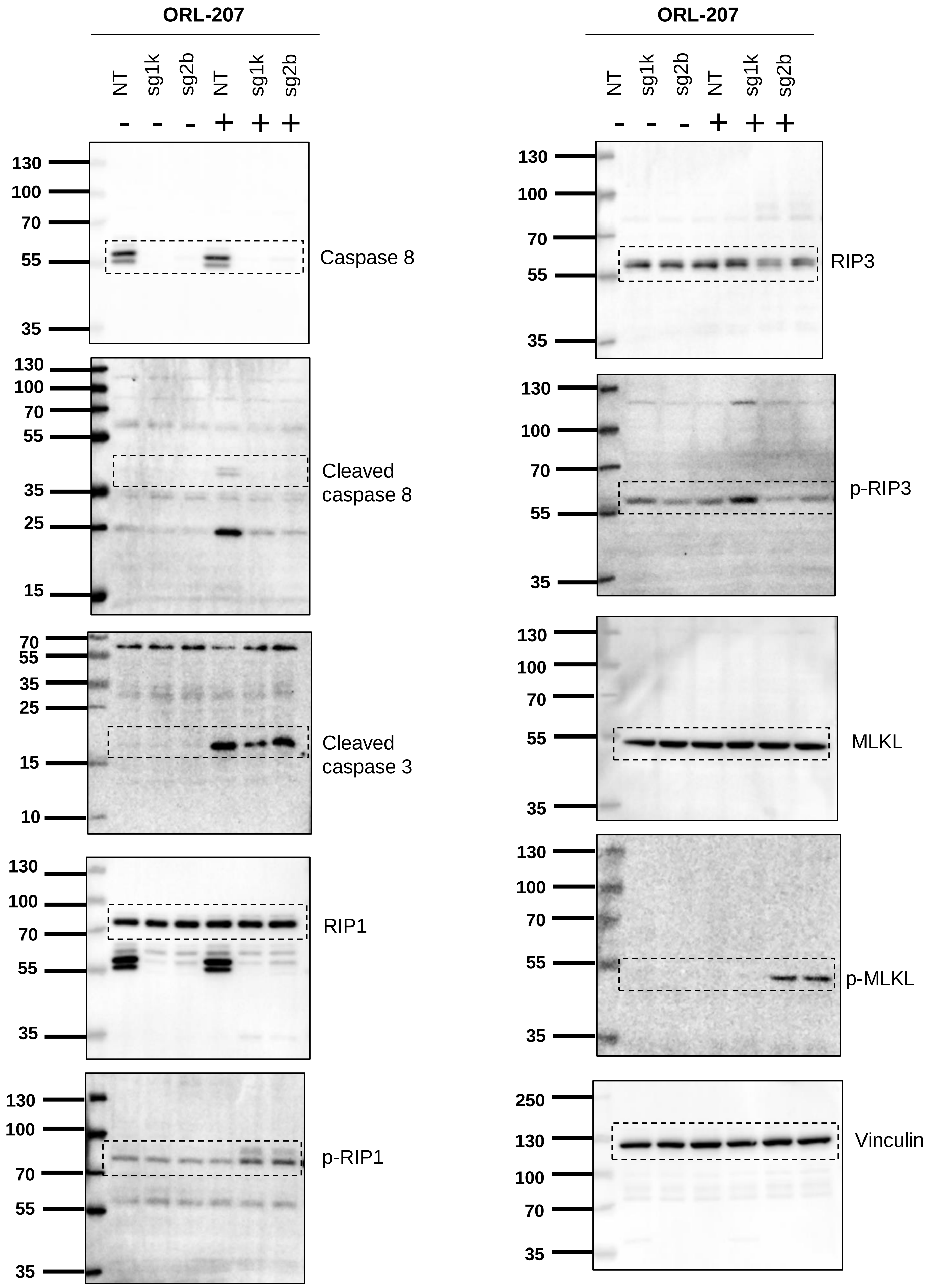

Supplementary Figure 6 – All uncropped western blot images

For Supp. Fig. 5B

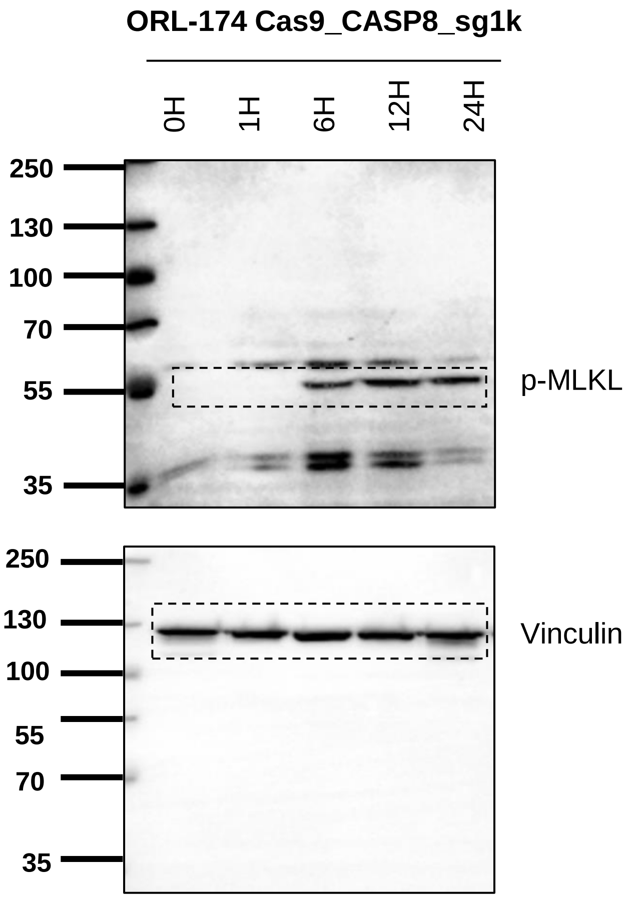

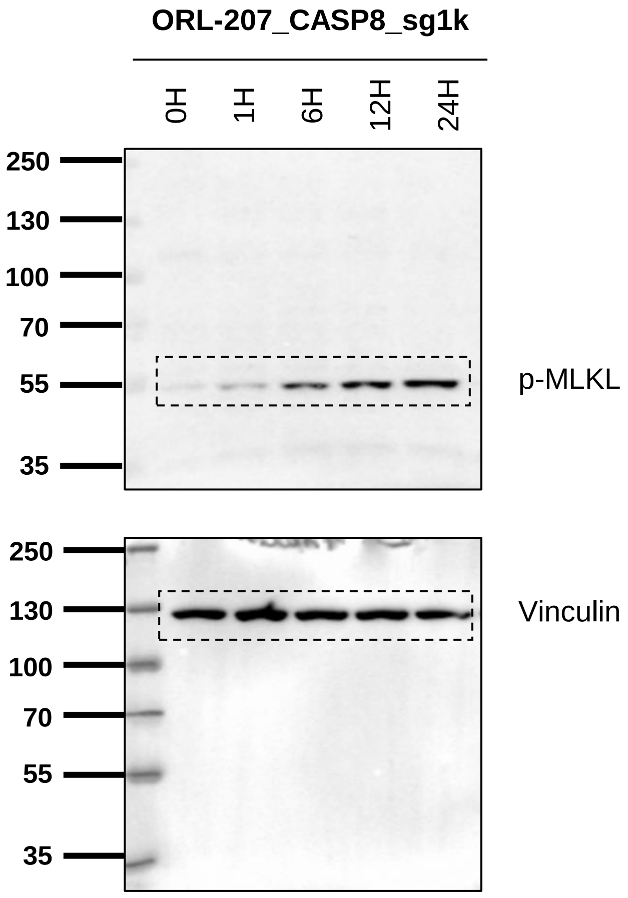

Supplementary Figure 6 – All uncropped western blot images

For Supp. Fig. 5C

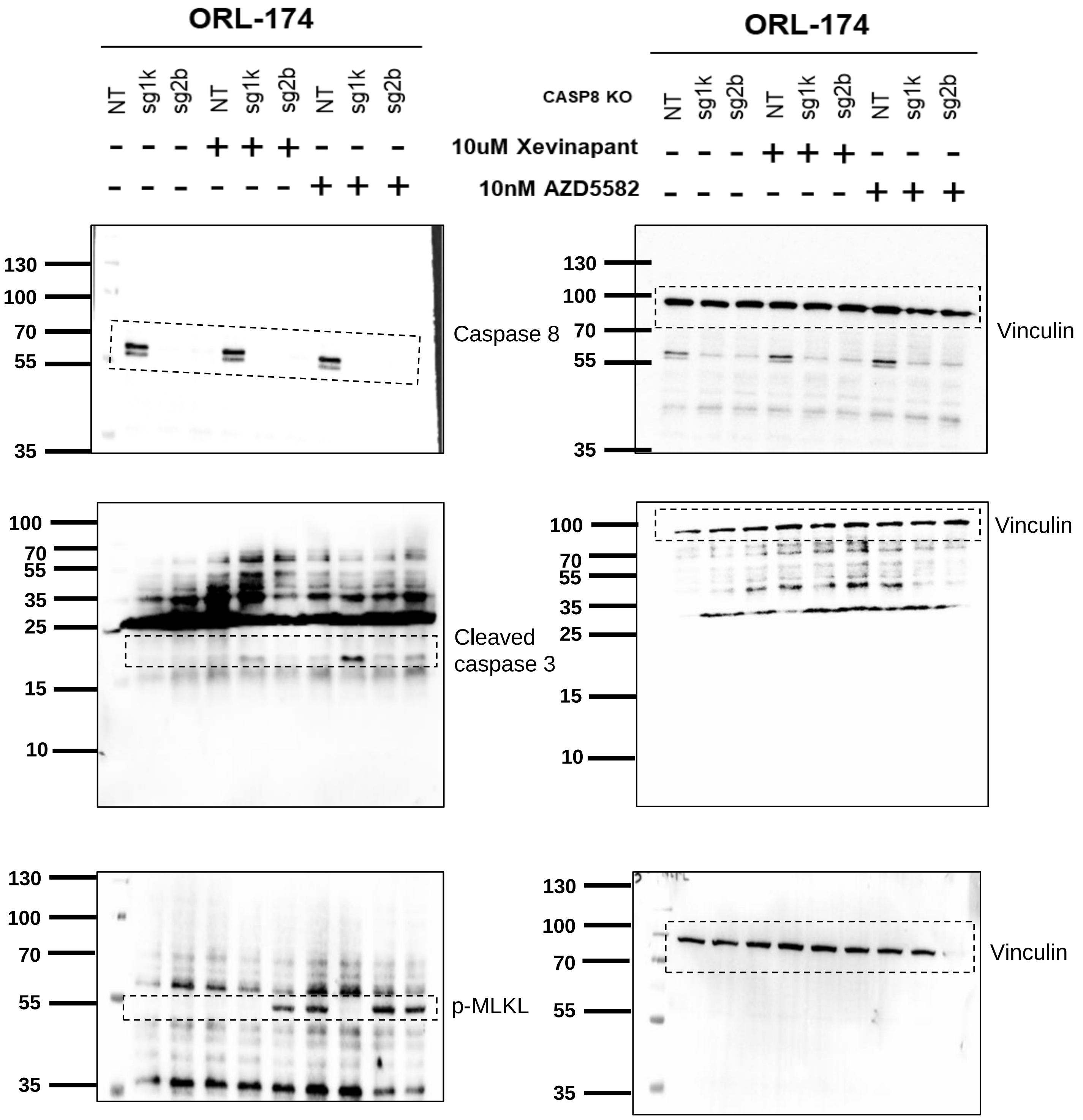

Supplement: Supplementary Figure 6 — All uncropped western blot images [file crc-24-0136_supplementary_figure_6_suppsf6.pdf]
